# Phyllostictine A: total synthesis, structural verification and determination of substructure responsible for plant growth inhibition†
†Electronic supplementary information (ESI) available: Experimental procedures and characterisation data for all new compounds, copies of ^1^H and ^13^C NMR spectra, XRD data for **5**, chiral HPLC analysis of **9** and root growth inhibition assays. CCDC 1838655. For ESI and crystallographic data in CIF or other electronic format see DOI: 10.1039/c8cc03349h


**DOI:** 10.1039/c8cc03349h

**Published:** 2018-06-13

**Authors:** Martin Riemer, Veselina V. Uzunova, Nastja Riemer, Guy J. Clarkson, Nicole Pereira, Richard Napier, Michael Shipman

**Affiliations:** a Department of Chemistry , University of Warwick , Gibbet Hill Road , Coventry , CV4 7AL , UK . Email: m.shipman@warwick.ac.uk; b School of Life Sciences , University of Warwick , Gibbet Hill Road , Coventry , CV4 7AL , UK

## Abstract

The first total synthesis of phyllostictine A (PA) is reported, which confirms the structure of this fungal metabolite and its (6*S*,7*R*,8*S*)-stereochemistry. Both synthetic PA and an analogue containing the 5-methylene-1,5-dihydro-2*H*-pyrrol-2-one nucleus exhibit μM inhibitory activity in root growth assays against *Arabidopsis thaliana*, indicating that this heterocyclic subunit is key to the herbicidal activity of the natural product.

## Abstract 2

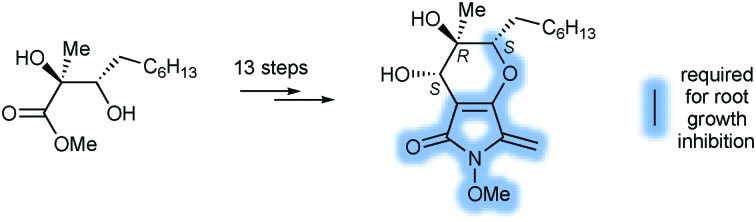
The first total synthesis of phyllostictine A is reported and evidence presented that the heterocyclic subunit is the key to the μM herbicidal activity.

To address the rising levels of resistance emerging against existing crop protection agents, there is an urgent need to discover and develop new herbicides with novel mechanisms of action.^1^ In this search, natural products with their unique chemical architectures and prominent bioactivities offer an excellent entry point.^2^ In 2008, Evidente *et al.* reported the isolation and structural elucidation of a new class of natural herbicide produced by the fungus *Phyllosticta cirsii.*^3^ Four compounds named phyllostictines A–D were identified of which the most potent was phyllostictine A (PA). PA displays considerable efficacy in leaf puncture assays on Canada thistle, as well as against isolated protoplasts.^4^ PA is much more potent than fusaric acid, a well-known and powerful toxin, and faster acting than glyphosate. Thus, it represents a potentially interesting lead in the development of new herbicides.

Evidente *et al.*^3^ assigned an oxazatricycloalkenone ring system to these metabolites (Fig. 1), and they subsequently attracted attention as targets for synthesis.^5^ However, as part of investigations into the biosynthetic origin of PA, Trenti and Cox revised the core structure of the phyllostictines to a bicyclic 3-methylene tetramic acid (Fig. 1).^6^ Moreover, they recognised that phyllostictine B is spectroscopically identical to phaeosphaeride A previously isolated by Clardy from an endophytic fungus.^7^ Since the stereochemical configuration of this natural product had been unambiguously established by total synthesis of *ent*-phaeosphaeride A,^8^ and by X-ray diffraction,^9^ Cox was able to assign the relative and absolute configuration of phyllostictine B.^6^ By analogy, the (6*S*,7*R*,8*S*)-stereochemistry for PA, a hexaketide containing two additional methylenes in the alkyl side chain was proposed (Fig. 1).

**Fig. 1:**
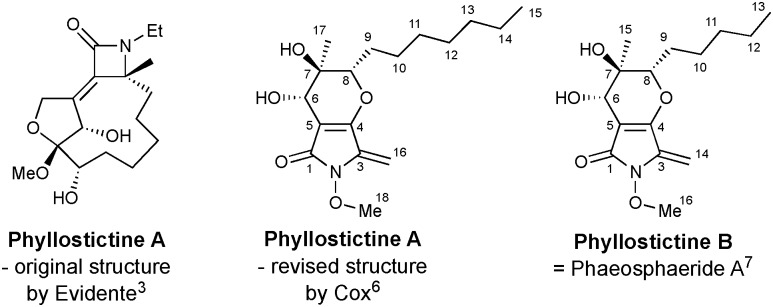
Chemical structures of phyllostictine A and B with atom numbering.

In this communication, the first total synthesis of PA is achieved confirming the structure revisions put forward by Trenti and Cox in 2017.^6^ Additionally, using simplified analogues based on the 5-methylene-1,5-dihydro-2*H*-pyrrol-2-one nucleus, the structural basis for the herbicidal activity of PA is revealed.

The presence of a 5-methylene-1,5-dihydro-2*H*-pyrrol-2-one within the revised structure of PA suggested that this heterocyclic scaffold might play a key role in its bioactivity. To test this hypothesis, and to develop chemical routes to this framework, a synthetic route to **1** was initially devised. This was conveniently achieved in 5 steps from dimethyl acetylene dicarboxylate, **2** (Scheme 1). First, conjugated addition of MeOH to **2** in presence of a catalytic amount of *n*-BuLi afforded **3** in 67% yield. Careful temperature control was necessary to achieve the high (>20 : 1) *E* : *Z* selectivity. Conversion to *N*-methoxymaleimide **4** involved saponification and treatment with methoxyamine under modified Steglich conditions. Addition of MeMgBr to **4** provided alcohol **5** in 79% yield by way of regiocontrolled addition at C-2. This regiochemical outcome was verified by single crystal X-ray diffraction (XRD).‡
‡Crystal data for **5**: C_7_H_11_NO_4_ (*M* = 173.17 g mol^–1^), monoclinic, space group *P*2_1_/*n* (no. 14), *a* = 9.7222(3) Å, *b* = 7.91107(17) Å, *c* = 11.6743(3) Å, *β* = 102.086(3)°, *V* = 878.00(4) Å^3^, *Z* = 4, *T* = 150(2) K, *μ*(CuKα) = 0.923 mm^–1^, *D*_calc_ = 1.310 g cm^–3^, 6016 reflections measured (13.31° ≤ 2*Θ* ≤ 156.668°), 1844 unique (*R*_int_ = 0.0245, *R*_sigma_ = 0.0198) which were used in all calculations. The final *R*_1_ was 0.0382 (*I* > 2*σ*(*I*)) and w*R*_2_ was 0.1115 (all data). Data deposited at Cambridge Crystallographic Data Centre: CCDC 1838655.†
 No evidence for addition to the other amide carbonyl group was observed even using excess Grignard reagent (10 equiv.). Finally, treatment with TFA induced efficient elimination to **1**.

**Scheme 1:**
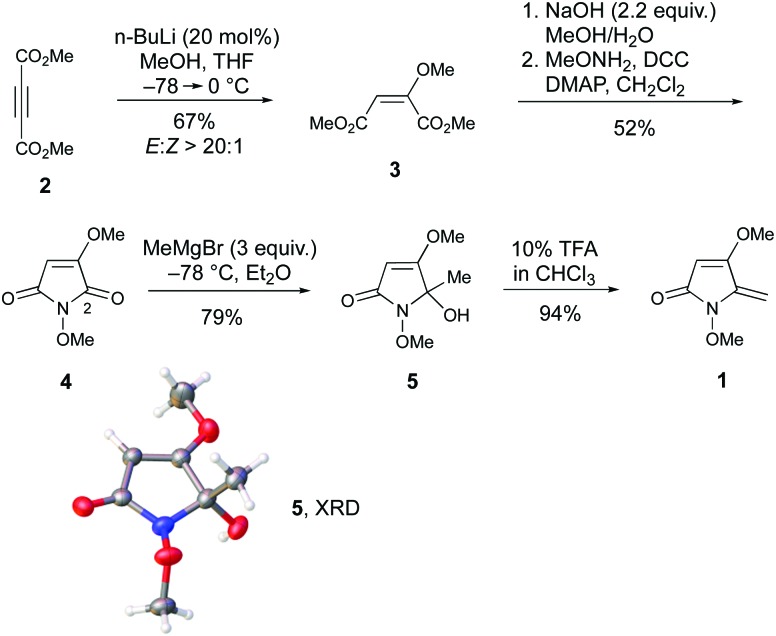
Synthesis of 5-methylene-1,5-dihydro-2*H*-pyrrol-2-one **1**.

Next, we embarked on the total synthesis of PA to resolve the structural uncertainties, and to access quantities of the natural product for herbicidal screening. To expedite this work, we adopted a strategy analogous to that used by Kobayashi *et al.* for the synthesis of *ent*-phaeosphaeride A,8*a* which possesses the opposite absolute configuration and a shorter alkyl side chain. To initiate the synthesis, access to multi-gram quantities of (2*S*,3*S*)-**9** was required (Scheme 2). First, the Still–Gennari reagent **6** was reacted with octanal in a Horner–Wadsworth–Emmons reaction to give methyl ester **7** in 90% yield as the *Z*-stereoisomer (*Z* : *E*; >99 : 1).^10^ Lower yields (67%) were observed when the aldehyde was not freshly distilled prior to use. Subjection of **7** to Sharpless asymmetric dihydroxylation^11^ using AD-mix-α provided diol (2*S*,3*S*)-**8** in 84% yield. Excellent enantiocontrol (98% ee) was observed as established by chiral HPLC analysis after conversion into (2*S*,3*S*)-**9**. The assignment of the absolute configuration of **8** was made using the Sharpless mnemonic which is known to work reliably for angelic esters.^12^ Consistent with Kobayashi's studies on the synthesis of *ent*-phaeosphaeride A,8*a* the selective benzylation of **8** proved challenging. Using 2,4,6-tris(benzyloxy)-1,3,5-triazine (TriBOT) and TfOH,^13^ monobenzylated **9**§
§**9** contained traces (*ca.* 15%) of the isomer (R^1^ = Bn; R^2^ = H) in which the tertiary alcohol has been selectively benzylated. This impurity was removed by chromatography after reduction to **11**. was obtained in 55% yield alongside bis-benzylated **10** (15%) and unreacted diol **8** (30%). These products were separable and by debenzylating **10** through catalytic hydrogenation, it was possible to regenerate additional quantities of diol **8** which could be resubjected to the TriBOT conditions. In this way, the conversion of **8** to **9** could be improved to 82% after two rounds of recycling (see ESI†).

**Scheme 2:**
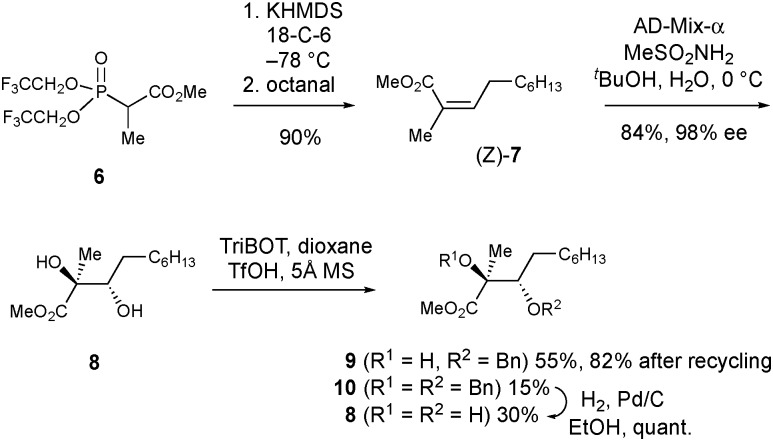
Asymmetric synthesis of (2*S*,3*S*)-**9**.

To complete the synthesis of PA from **9**, a 12-step sequence was used to consecutively construct the dihydropyran and pyrrolidine rings. The sequence followed that used by Kobayashi *et al.* to make *ent*-phaeosphaeride A,8*a* although modification of some steps was required (Scheme 3).

**Scheme 3:**
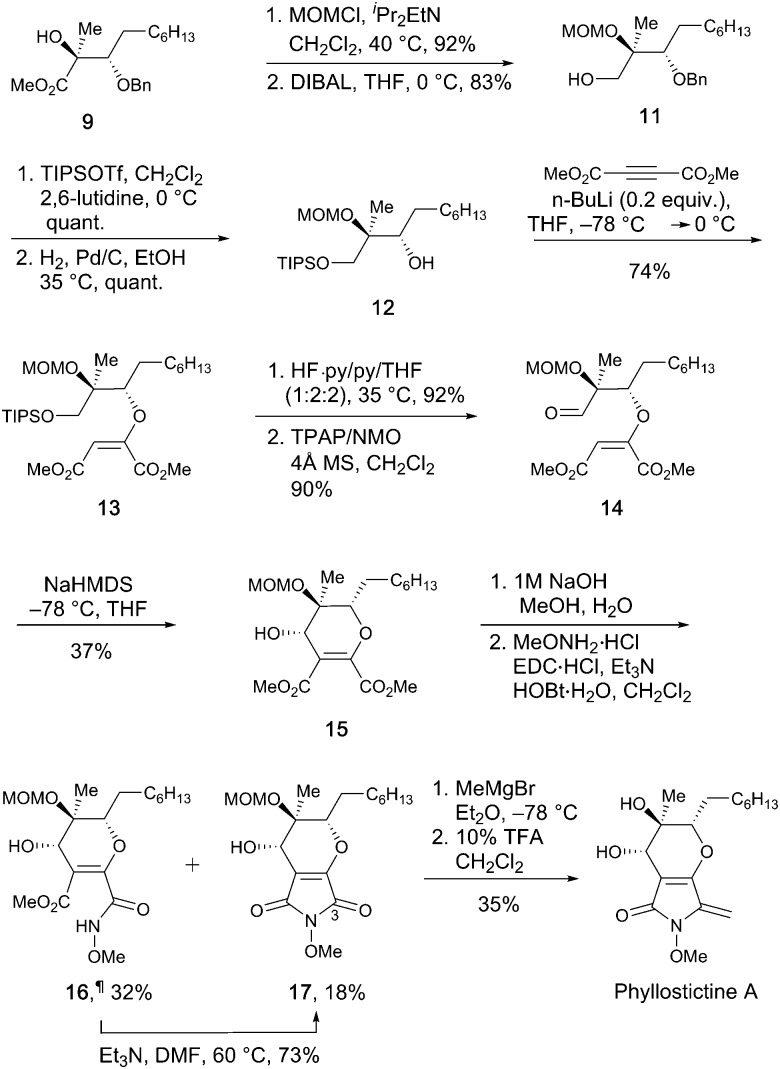
Total synthesis of phyllostictine A.

First, the hydroxyl group of **9** was protected as the MOM ether, then the ester group reduced with DIBAL to give alcohol **11** in good overall yield. Protection of the resultant primary alcohol as its TIPS ether, followed by Pd/C catalysed hydrogenolysis provided secondary alcohol **12**. Conjugated addition of **12** to DMAD in the presence of a catalytic amount of *n*-BuLi gave **13** as the major isomer (*E* : *Z*; >20 : 1). As in the synthesis of **3**, careful temperature control was critical to achieve high *E* to *Z* selectivity. Other bases are reported to be less effective for closely related additions.8*d* Next, the TIPS-ether of **13** was cleaved with HF·py and the resulting alcohol oxidised to aldehyde **14** using TPAP.^14^ Stereocontrolled six-membered ring formation was achieved by vinyl anion aldol reaction with NaHMDS at –78 °C. The (*S*)-stereochemistry at the newly created secondary alcohol was deduced by the presence of a 2.7 Hz W-coupling.8*a* The *n*-heptyl and MOM ether groups most likely adopted pseudo-equatorial orientations during this ring closure, with the formyl group chelated to the sodium ion of the intermediate allenic enolate.8*a* To complete the synthesis, *N*-methoxymaleimide **17** was synthesised *via* saponification of **15** and treatment with methoxyamine under EDC/HOBt conditions. This led to formation of **17** alongside appreciable quantities of methyl ester **16**¶
¶The MeONH amide and methyl ester groups within **16** may be transposed. The regiochemical assignment was tentatively made based on the basis of the relative reactivity of the C=O groups within **4**. in which the final ring closure had not occurred. Simply warming **16** in Et_3_N/DMF enabled amide bond formation providing additional quantities of **17**. Using this sequence, **15** was converted to **17** in 41% overall yield. Finally, controlled addition of MeMgBr to C-3 of **17** followed by dehydration and MOM deprotection with TFA provided phyllostictine A in 35% over the two steps after purification by preparative TLC then reverse-phase HPLC. This final addition/elimination sequence was less efficient than for the conversion of **4** to **1** (Scheme 1), however supplies of **17** were limited preventing further optimization.

Verification of the completion of the first total synthesis of PA was confirmed by comparison of the ^13^C and ^1^H NMR spectra in d_6_-DMSO of our synthetic material with the data reported by Cox for the natural product,^6^ and with those reported for *ent*-phaeosphaeride A,8*a* with excellent agreement between these data sets (Table 1). Moreover, the specific optical rotation of the natural product ([*α*]25D = –87.5 (*c* 0.2, CHCl_3_))^1^ and our synthetic material ([*α*]21D = –83.3 (*c* 0.3, CHCl_3_)) are in close agreement. Since the absolute configuration of our synthetic material was established using the Sharpless AD reaction, we confidently assign the (6*S*,7*R*,8*S*)-configuration to the natural product.

**Table 1:** Comparison of ^1^H and ^13^C NMR data for synthetic and natural phyllostictine A and *ent*-phaeosphaeride A in d_6_-DMSO

Atom	Phyllostictine				Atom	*ent*-Phaeosphaeride A8*a*	
	Natural (Cox^6^)		Synthetic (this work)				
	*δ*C/ppm^*a*^	*δ*H/ppm^*b*^ ^,^^*c*^	*δ*C/ppm^*d*^	*δ*H/ppm^*c*^ ^,^^*e*^		*δ*C/ppm^*d*^	*δ*H/ppm^*c*^ ^,^^*e*^
1	166.4		166.5		1	166.5	
3	137.1		137.1		3	137.1	
4	155.3		155.3		4	155.3	
5	104.8		104.8		5	104.8	
6	64.4	3.86, d, *J* = 5.6 Hz	64.3	3.79, s	6	64.3	3.87, d, *J* = 5.5 Hz
7	70.6		71.0		7	70.9	
8	86.2	4.06, m	86.3	4.00, d, *J* = 11.3 Hz	8	86.2	4.07, d, *J* = 11.0 Hz
9	27.5	1.82, m	27.6	1.87–1.77, m	9	27.6	1.82, m
		1.55, m		1.56–1.49, m			1.57–1.39, m
10	26.3	1.44, m	26.5	1.46–1.40, m	10	26.1	1.57–1.39, m, 2H
		1.33, m		1.35–1.30, m			
11	31.1	1.26, m	31.2	1.26, m	11	30.9	1.36–1.10, m, 2H
		1.19, m		1.19, m			
12	21.9	1.24, m	22.1	1.24, m	12	21.9	1.36–1.10, m, 2H
13	28.5	1.25, m	28.7	1.25, m			
14	28.4	1.25, m	28.6	1.25, m			
15	13.8	0.85, t, *J* = 6.8 Hz, 3H	14.0	0.79, t, *J* = 6.7 Hz, 3H	13	13.8	0.86, t, *J* = 6.5 Hz, 3H
16	90.5	4.95, d, *J* = 1.5 Hz	90.9	4.96, s	14	90.7	4.97, s, 2H
		4.96, d, *J* = 1.5 Hz		4.96, s			
17	20.2	1.17, s, 3H	19.9	1.11, s, 3H	15	20.3	1.18, s, 3H
18	63.6	3.78, s, 3H	63.8	3.72, s, 3H	16	63.7	3.79, s, 3H
6-OH		5.34, d, *J* = 5.9 Hz		5.37, s	6-OH		5.42, d, *J* = 5.5 Hz
7-OH		4.84, s		4.85, s	7-OH		4.90, s

^*a*^100 MHz.

^*b*^400 MHz.

^*c*^Integrate as 1H unless otherwise stated.

^*d*^125 MHz.

^*e*^500 MHz.

To determine which elements of the natural product are needed for herbicidal activity, root growth inhibition assays^15^ were performed using *Arabidopsis thaliana* seedlings treated with synthetic PA, **1** and **4**. After vernalisation for 2 days, seeds were germinated at 22 °C and grown for 6 days before seedlings were transferred to fresh plates incorporating 8 different concentrations of test compound. Elongation of the primary root was assessed after exposure to inhibitor for 6 days, from which dose response curves and IC_50_ values were derived (see ESI†). Using this assay, the following IC_50_ values were determined: synthetic PA: 9 ± 1 μM; **1**: 35 ± 6 μM; **4**: 205 ± 19 μM; glyphosate: 7 ± 2 μM. The dose–response curves for PA, **1** and **4** are provided in Fig. 2. The following conclusions can be drawn from this data: (i) PA possesses similar potency to the ubiquitous herbicide glyphosate in this well-established herbicidal assay; (ii) simplified analogue **1** is only 4× less potent than the natural product, suggesting that this substructure is important for root growth inhibition; (iii) removal of the exocyclic double bond as in **4** leads to a significant reduction in potency. This finding is consistent with observations that PA conjugates with glutathione,^16^ and suggests that it may act as a Michael acceptor through the exocyclic double bond.^17^

**Fig. 2:**
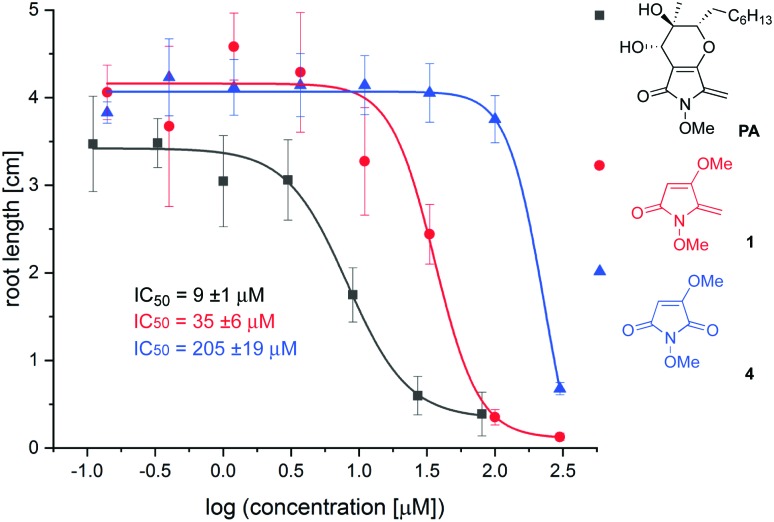
Root growth inhibition assays.

In summary, the first total synthesis of phyllostictine A was achieved in 15 steps from octanal, which has confirmed the gross structure and absolute stereochemistry of this natural product. Additional studies led to the identification of simple heterocyclic derivative **1**, which can be made in just 5 steps from readily available starting materials, yet retains much of the herbicidal activity of the natural product. In future work, we will seek to explore the details of the mechanism of action of PA, and the potential of related analogues for crop protection.

The support of the EPSRC (EP/K031783/1), BBSRC and EU is gratefully acknowledged. M. R. was supported by a Marie-Sklodowska-Curie Individual Fellowship (MSCA-IF-EF-4887, Project 705079). We thank Professor Russell Cox (Leibniz Universität Hannover) for helpful communications regarding the structural reassignment of PA. Funding for Gold open-access publishing was provided by Research Councils UK.

## Conflicts of interest

There are no conflicts to declare.

## Supplementary Material

Supplementary information

Crystal structure data
