# Supplementary material for: Phyllostictine A: total synthesis, structural verification and determination of substructure responsible for plant growth inhibition
Source: Chem Commun (Camb). 2018 Jun 13;54(52):7211–4. doi: 10.1039/c8cc03349h (PMC6018569; doi:10.1039/c8cc03349h)
Supplement: Supplementary file 1 [file CC-054-C8CC03349H-s001.pdf]

Electronic Supplementary Material (ESI) for ChemComm.
This journal is © The Royal Society of Chemistry 2018

Phyllostictine A: Total Synthesis, Structural Verification and Determination of Substructure Responsible for Plant Growth Inhibition

Martin Riemer,[a] Veselina Uzunova,[b] Nastja Riemer,[a] Guy J. Clarkson,[a] Nicole Pereira,[b] Richard Napier[b] and Michael Shipman*,[a]

[a] Department of Chemistry, University of Warwick, Gibbet Hill Road, Coventry, CV4 7AL, U.K. Fax: +44 2476 524112; Tel: +44 2476 523186; E-mail: m.shipman@warwick.ac.uk

[b] School of Life Sciences, University of Warwick, Gibbet Hill Road, Coventry, CV4 7AL, U.K.

Electronic Supplementary Information

1. General details S2

2. Experimental procedures and characterisation data S3-S18

3. Copies of ^{1}H and ^{13}C NMR spectra S19-S37

4. X-ray data for **5** S38

5. Plant growth assays for **1**, **4** and synthetic phyllostictine A S39

6. References S40

1. General details

Anhydrous solvents were purchased from Sigma-Aldrich or Acros Organics in Sure-Seal™ bottles for use as reaction solvents. All other solvents were reagent grade and used as received. Petroleum ether refers to the fraction that boils in the range 40-60 °C. Commercially available starting materials were used without purification unless otherwise stated.

Thin layer chromatography was performed on pre-coated aluminium-backed plates (Merck Silica gel 60 F254), visualised by UV 254 nm and then stained with phosphomolybdic acid (PMA) dip. Flash column chromatography was performed using Aldrich 40-63 μm silica gel.

Nuclear magnetic resonance (NMR) spectra were recorded on Bruker DPX (300 or 400 MHz), or AV (500 MHz) spectrometers. Chemical shifts (δ) are reported in parts per million (ppm) relative to the solvent residual peaks ($CDCl_3$ δ_H: 7.26 ppm, δ_C: 77.16 ppm; DMSO-d_6 δ_H: 2.50 ppm, δ_C 39.52 ppm). Coupling constants (*J*) are reported in hertz (Hz). Splitting patterns are abbreviated as follows: singlet (s), doublet (d), triplet (t), quartet (q), multiplet (m), broad (b), or combination of these. NMR assignments were deduced using 2D experiments (COSY, HSQC and HMBC).

Low-resolution mass spectra were recorded on an Agilent Technologies 6130 Quadrupole LC-MS instrument. High-resolution mass spectra were recorded using a Bruker MaXis Impact. Infrared spectra were recorded with a Bruker ALPHA Platinum ATR apparatus. Optical rotations $[\alpha]_D^T$ were measured using an AA-1000 polarimeter. Melting points were recorded with a Gallenkamp MPD350 melting point apparatus.

2. Experimental procedures and characterisation data

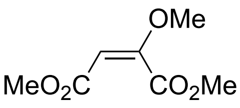

3

Dimethyl 2-methoxymaleate, 3.[1,2] To a solution of MeOH (480 mg, 15.0 mmol) in THF (50.0 mL) was added *n*-BuLi (2.0 M, in *n*-hexane, 20 mol%, 1.50 mL) at –78 °C. The reaction mixture was stirred for 15 minutes at this temperature. A solution of dimethyl acetylenedicarboxylate (freshly distilled, 2.13 g, 15.0 mmol) in THF (10.0 mL) was added dropwise via syringe pump over 40 minutes. The reaction mixture was slowly allowed to warm up to 0 °C over ca 4 h. The reaction was quenched with a solution of saturated NH_4Cl solution and brine (1:1, 20 mL). The aqueous phase was extracted with ethyl acetate (3 x 100 mL). The combined organic phases were dried over $MgSO_4$ and concentrated *in vacuo*. The crude product was purified by column chromatography on silica (petroleum ether:EtOAc; 10:1) to afford **3** (1.76 g, 67%) as a white solid. **Mp** 41 – 42 °C (Lit[2]: 40 °C); **^{1}H-NMR** (400 MHz, $CDCl_3$) δ 5.20 (s, 1H, CH), 3.88 (s, 3H, C=C-OCH_3), 3.74 (s, 3H, CO_2CH_3), 3.70 (s, 3H, CO_2CH_3); **^{13}C NMR** (101 MHz, $CDCl_3$) δ 166.4 (C=O), 164.1 (O*C*=C), 162.6 (C=O), 93.2 (CH), 57.1 (CO_2*C*H_3), 53.1 (C=C-O*C*H_3), 51.8 (CO_2*C*H_3); **IR** (film) 2955, 2072, 1878, 1715, 1433, 1142, 691 cm^{-1}; **MS** (ESI+) *m/z* 197 $[M+Na]^+$; **HRMS** (ESI+) Calcd for $C_7H_{10}NaO_5^+$ $[M+Na]^+$: 197.0420, found 197.0421. ^{1}H and ^{13}C data identical to those reported previously.[2]

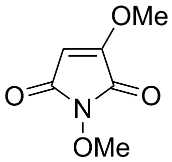

4

1,3-Dimethoxy-1H-pyrrole-2,5-dione, **4**. To a solution of **3** (696 mg, 4.0 mmol) in MeOH (50 mL) was added aqueous 1M NaOH (8.8 mL). The reaction mixture was stirred at 35 °C for 6 h (conversion monitored by NMR in D_2O until >95% conversion). The reaction mixture was concentrated *in vacuo* then acidified by addition of aqueous 2M HCl (10 mL). The mixture was extracted with ethyl acetate (10 x 50 mL), then the combined organic phases concentrated *in vacuo*. To the crude acid (610 mg) in CH_2Cl_2 (20 mL) were added successively $MeONH_2$ (376 mg, 8.0 mmol containing 10% of *N*,*N*-diisopropylethylamine),[3] *N*,*N'*-dicyclohexylcarbodiimide (1.81 g, 8.8 mmol) and 4-dimethyl-aminopyridine (98 mg, 0.8 mmol). The reaction mixture was stirred overnight at room temperature then concentration *in vacuo*. Purification by column chromatography (petroleum ether:EtOAc; 2:1→1:2) gave **4** (327 mg, 52%) as a white solid. **Mp** 104 – 106 °C; **^{1}H-NMR** (400 MHz, $CDCl_3$) δ 5.32 (s, 1H, CH), 3.95 (s, 3H, OCH_3), 3.93 (s, 3H, OCH_3); **^{13}C NMR** (101 MHz, $CDCl_3$) δ 165.8 (C=O), 161.4 (C=O), 158.4 (O*C*=C), 94.3 (CH), 66.0 (OCH_3), 58.7 (OCH_3); **IR** (film) 3106, 2950, 1727, 1636, 1325, 1096, 8213 cm^{-1}; **MS** (ESI+) *m/z* 180 $[M+Na]^+$; **HRMS** (ESI+) Calcd for $C_6H_7NNaO_4^+$ $[M+Na]^+$: 180.0267, found 180.0268.

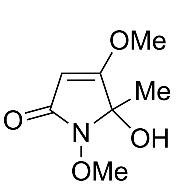

5

5-Hydroxy-1,4-dimethoxy-5-methyl-1,5-dihydro-2H-pyrrol-2-one, 5. Dione **4** (90 mg, 0.57 mmol) was dissolved in Et_2O (20.0 mL). A solution of MeMgBr (3.0 M in Et_2O, 0.57 mL, 1.71 mmol) was added dropwise at –78 °C. The reaction mixture was stirred until complete consumption of the starting material (by TLC, ca. 25 min) then quenched with saturated NH_4Cl solution (10.0 mL). The aqueous phase was extracted with ethyl acetate (3 x 30 ml). The combined organic phases were dried over Na_2SO_4 and concentrated *in vacuo*. The crude material was purified by column chromatography (EtOAc) to give **5** (78 mg, 79%) as a white solid. **Mp** 109 – 111 °C; **^{1}H-NMR** (400 MHz, acetone-d_6) δ 5.44 (s, 1H, O*H*), 4.95 (s, 1H, CH), 3.84 (s, 3H, $COCH_3$), 3.83 (s, 3H, $NOCH_3$), 1.46 (s, 3H, C-CH_3); **^{13}C NMR** (101 MHz, acetone-d_6) δ 176.4 (*C*OCH_3), 170.1 (C=O), 90.7 (CH), 88.6 (COH), 65.7 ($NOCH_3$), 58.6 (CO*C*H_3), 21.7 (CH_3); **IR** (film) 3301, 2944, 1880, 1700, 1626, 1068, 692 cm^{-1}; **MS** (ESI+) *m/z* 196 $[M+Na]^+$; **HRMS** (ESI+) Calcd for $C_7H_{11}NNaO_4^+$ $[M+Na]^+$: 196.0580, found 196.0581.

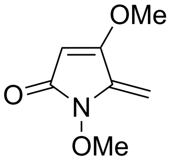

1

1,4-Dimethoxy-5-methylene-1,5-dihydro-2H-pyrrol-2-one, 1. Alcohol **5** (53 mg, 0.31 mmol) was dissolved in a solution of TFA in CH_2Cl_2 (10% TFA, 1 mL) and stirred at room temperature for 30 min. The reaction mixture was concentrated *in vacuo* and the crude material purified by column chromatography (petroleum ether:EtOAc; 1:1) to give **1** (45 mg, 94%) as a colourless oil. **^{1}H-NMR** (500 MHz, acetone-d_6) δ 5.19 (s, 1H, CH), 4.98 (s, 1H, CH_2-H_{trans}), 4.93 (s, 1H, CH_2-H_{cis}), 3.91 (s, 3H, $COCH_3$), 3.84 (s, 3H, $NOCH_3$); **^{13}C NMR** (126 MHz, acetone-d_6) δ 167.4 (C=O), 163.8 (*C*OCH_3), 139.7 (*C*=CH_2), 92.2 (CH), 90.3 (CH_2), 64.1 ($NOCH_3$), 58.5 (CO*C*H_3); **IR** (film) 3109, 2986, 1714, 1600, 1441, 1107, 980 cm^{-1}; **MS** (ESI+) *m/z* 178 $[M+Na]^+$; **HRMS** (ESI+) Calcd for $C_7H_9NO_3Na$ $[M+Na]^+$: 178.0475, found 178.0475.

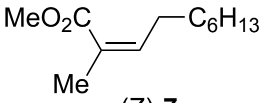

(Z)-**7**

Methyl (*Z*)-2-methyldec-2-enoate, 7.[4] A solution of KHMDS (1.0 M in THF, 4.75 mL, 4.75 mmol) was added dropwise to a stirred solution of methyl 2-(bis(2,2,2-trifluoroethoxy)phosphoryl)propanoate[5] (**6**) (1.73 g, 5.00 mmol) and 18-crown-6 (1.28 g, 4.85 mmol) at –78 °C and stirring was continued for 20 minutes. A solution of freshly distilled octanal (608 mg, 4.75 mmol) in THF (10.0 mL) was added dropwise to the reaction mixture. After stirring for 1.5 h, the mixture was quenched with saturated solution of NH_4Cl (20 mL) and allowed to warm to room temperature. The mixture was extracted with ethyl acetate (3 x 100 mL), the combined organic phases dried over $MgSO_4$ then concentrated *in vacuo*. The crude product was purified by column chromatography (petroleum ether:Et_2O; 100:1) to give (*Z*)-**7** (850 mg, 90%) as a colourless oil. **^{1}H-NMR** (400 MHz, $CDCl_3$) δ 5.93 (tq, *J* = 7.4, 1.3 Hz, 1H, C*H*=C), 3.73 (s, 3H, CO_2CH_3), 2.44 (td, *J* = 7.6, 1.1 Hz, 2H, CH_2-CH=C), 1.89 (d, *J* = 1.3 Hz, 3H, CH=C-CH_3), 1.42 – 1.34 (m, 2H, CH_2), 1.33 – 1.24 (m, 8H, 4x CH_2), 0.88 (t, *J* = 6.8 Hz, 3H, CH_2-CH_3); **^{13}C-NMR** (101 MHz, $CDCl_3$) δ 168.7 (*C*O_2Me), 144.0 (*C*H=C), 126.8 (CH=*C*), 51.3 (CO_2CH_3), 32.0 (CH_2), 29.8 (CH_2), 29.6 (CH_2), 29.5 (CH_2), 29.3 (CH_2), 22.8 (CH_2), 20.8 (C=C-*C*H_3), 14.2 (C_6H_{12}-*C*H_3); **IR** (film) 2955, 2927, 2857, 1720, 1460, 1259 cm^{-1}. Data identical to those reported previously.[4]

The reaction was performed under the same conditions but on four times the scale, KHMDS (18.7 mL), methyl 2-(bis(2,2,2-trifluoroethoxy)phosphoryl)propanoate[5] (**6**) (6.83 g, 19.7 mmol), octanal (2.40 g, 18.7 mmol) and 18-crown-6 (5.04 g, 20.5 mmol) to obtain (*Z*)-**7** (3.35 g, 89%).

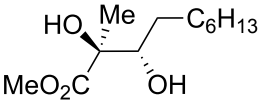

8

Methyl (2*S*,3*S*)-2,3-dihydroxy-2-methyldecanoate, 8. Methanesulfonamide (1.52 g, 16.0 mmol) and AD-Mix-α (21.3 g) were dissolved in ^{t}BuOH (50.0 mL) and water (50.0 mL) at room temperature. The reaction mixture was cooled to 0 °C and a solution of (*Z*)-**7** (4.20 g, 21.2 mmol) in MeCN (3.00 mL) and ^{t}BuOH (3.00 mL) was added dropwise. The reaction mixture was stirred at 0°C for 16 h. The reaction mixture was allowed to warm up to ambient temperature and stirred for 0.5 h at the same temperature after addition of Na_2SO_3 (25 g). The reaction mixture was extracted with ethyl acetate (3 x 100 mL). The combined organic phases were washed with aq 1M NaOH (20 mL) and brine (2 x 20 mL), dried over $MgSO_4$ and concentrated *in vacuo*. The crude product was purified by column chromatography (petroleum ether:EtOAc; 3:1 → 2:1) to give (2*S*,3*S*)-**8** (4.13 g, 84%) as a colourless oil. $[\alpha]_D^{32}$ −7.2 (*c* 0.5, $CHCl_3$); **^{1}H-NMR** (400 MHz, $CDCl_3$) δ 3.80 (s, 3H, CO_2CH_3), 3.58 (dd, *J* = 10.1, 1.6 Hz, 1H, C*H*OH), 3.48 (s, 1H, O*H*), 2.25 (s, 1H, O*H*), 1.64 – 1.46 (m, 2H, CH_2), 1.45 (s, 3H, C(OH)-CH_3), 1.32 – 1.24 (m, 10H, CH_2), 0.88 (t, *J* = 6.7 Hz, 3H, CH_2-CH_3); **^{13}C-NMR** (101 MHz, $CDCl_3$) δ 176.1 (CO_2Me), 77.4 (C(OH)-*C*(OH)-CO_2Me), 76.3 (*C*(OH)-C(OH)-CO_2Me), 52.9 (CO_2CH_3), 31.9 (CH_2), 31.9 (CH_2), 29.5 (CH_2), 29.3 (CH_2), 26.3 (CH_2), 22.8 (CH_2), 22.6 (C(OH)-CH_3), 14.2 (C_6H_{12}-CH_3); **IR** (film) 3467, 2953, 2924, 2856, 1732, 1456, 1246 cm^{-1}; **MS** (ESI+) *m/z* 254.8 $[M+Na]^+$; **HRMS** (ESI+) Calcd for $C_{12}H_{24}O_4Na$ $[M+Na]^+$: 255.1567, found 255.1566.

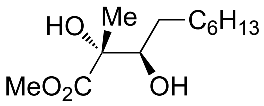

ent-**8**

Methyl (2*R*,3*R*)-2,3-dihydroxy-2-methyldecanoate, *ent*-8. Methanesulfonamide (27.0 mg, 0.28 mmol) and AD-Mix-β (254 mg) were dissolved in ^{t}BuOH (0.50 mL) and water (0.50 mL) at room temperature. The reaction mixture was cooled to 0 °C and a solution of (*Z*)-**7** (75.0 mg, 0.38 mmol) in MeCN (0.10 mL) and ^{t}BuOH (0.1 mL) was added dropwise. The reaction mixture was stirred at 0 °C for 16 h. The reaction mixture was allowed to warm up to ambient temperature and stirred for 0.5 h at the same temperature after addition of Na_2SO_3 (0.5 g). The reaction mixture was extracted with ethyl acetate (3 x 15 mL). The combined organic phases were washed with aq 1M NaOH (5 mL) and brine (2 x 5 mL), dried over $MgSO_4$ and concentrated *in vacuo*. The crude product was purified by column chromatography (petroleum ether:EtOAc; 3:1 → 2:1) to give (2*R*,3*R*)-**8** (46 mg, 52%) as a colourless oil. $[\alpha]_D^{32}$ +8.3 (*c* 0.3, $CHCl_3$); spectroscopic data as previously described.

Asymmetric dihydroxylation: determination of ee. The ee was determined by chiral HPLC of the benzylated product **9**, methyl (2*S*,3*S*)-2,3-dihydroxy-2-methyldecanoate and *ent*-**9**, methyl (2*R*,3*R*)-2,3-dihydroxy-2-methyldecanoate. **9** was analysed by chiral HPLC on an Agilent Technologies 1200 Series HPLC, using HPLC grade hexanes and propan-2-ol as the eluent, and detection by UV at 254 nm. Chiralcel OD column (0.46cm ø x 25 cm), 97:3 hexane:propan-2-ol, T = 25°C, flow rate = 0.5 mL/min. The "racemic" trace was produced by mixing quantities of the *R* and *S* enantiomers.

"racemic" mixture

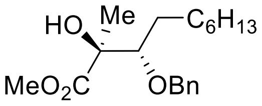

and

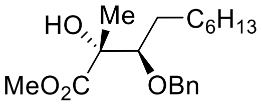

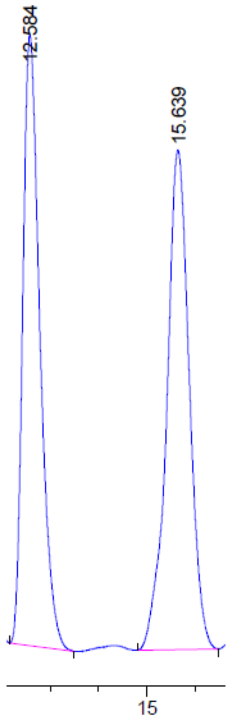

Peak #	RetTime [min]	Type	Width [min]	Area [mAU*s]	Height [mAU]	Area %
1	12.584	BB	0.3550	715.92059	29.20230	48.1654
2	15.639	BB	0.4031	770.46021	23.89963	51.8346
Totals :				1486.38080	53.10193	

Single enantiomer, **(2*S*,3*S*)-9**

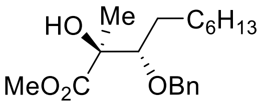

, 98% ee

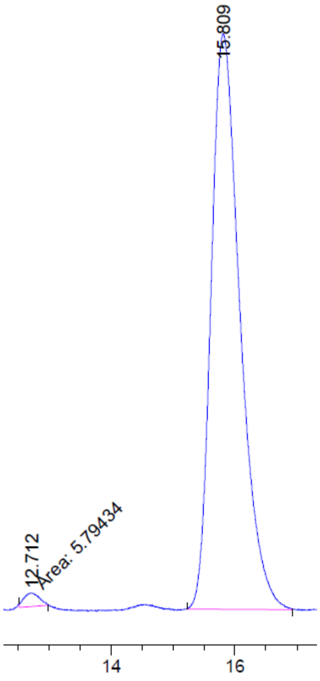

Peak #	RetTime [min]	Type	Width [min]	Area [mAU*s]	Height [mAU]	Area %
1	12.712	MM	0.2916	5.79434	3.31229e-1	1.2445
2	15.809	BV	0.4080	459.79474	14.19091	98.7555
Totals :				465.58908	14.52214	

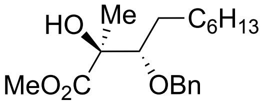

9

Methyl (2*S*,3*S*)-3-(benzyloxy)-2-hydroxy-2-methyldecanoate, 9. To a stirred solution of **8** (4.13 g, 17.8 mmol) in dioxane (135 mL) were added 5Å molecular sieve (3.6 g), 2,4,6-tris(benzyloxy)-1,3,5-triazine (2.60 g, 6.57 mmol) and dropwise TfOH (534 mg, 3.56 mmol). After stirring for 16 h at room temperature, the reaction was quenched by addition of saturated aq. $NaHCO_3$ solution (50 mL). The aqueous phase was extracted with ethyl acetate (3 x 100 mL), the combined organic phases dried over $MgSO_4$ and the solvent removed *in vacuo*. The crude product was purified by column chromatography (petroleum ether: EtOAc; 19:1 →2:1) to give: methyl (2*S*,3*S*)-2,3-bis(benzyloxy)-2-methyldecanoate (**10**) (1.12 g, 15%) as a yellow oil; **^{1}H-NMR** (400 MHz, $CDCl_3$) δ 7.31 – 7.17 (m, 10H, ArH), 4.51 – 4.48 (m, 4H, CH_2Ph), 3.75 (dd, *J* = 9.0, 2.9 Hz, 1H, CHOBn), 3.62 (s, 3H, CO_2CH_3), 1.44 (s, 3H, C(OBn)-CH_3), 1.32 – 1.12 (m, 12H, CH_2), 0.80 (d, *J* = 6.4 Hz, 3H, CH_2-CH_3); **9** (3.17 g, 55%, as mixture of isomers 5:1) as a yellow oil: $[\alpha]_D^{32}$ –6.3 (*c* 0.2, $CHCl_3$), NMR-data for the major isomer **^{1}H-NMR** (400 MHz, $CDCl_3$) δ 7.38 – 7.29 (m, 5H, ArH), 4.67 (s, 2H, CH_2Ph), 3.77 (s, 3H, CO_2CH_3), 3.52 (dd, *J* = 9.1, 2.0 Hz, 1H, CHOBn), 3.24 (s, 1H, OH), 1.44 (s, 3H, C(OH)-CH_3), 1.31 – 1.23 (m, 10H, CH_2), 1.21 (s, 2H, CH_2), 0.89 – 0.86 (m, 3H, CH_2-CH_3); **^{13}C-NMR** (101 MHz, $CDCl_3$) δ 175.9 (*C*O_2Me), 138.5 (*C*CH_2O), 128.5 (*C*H(Ar-o), 127.9 (*C*H(Ar-m)), 127.8 (*C*H(Ar-p), 85.0 (CH(OBn), 78.3 (COH), 75.0 (CH_2Bn), 52.8 (CO_2*C*H_3), 31.9 (CH_2), 31.1 (CH_2), 29.8 (CH_2), 29.4 (CH_2), 26.7 (CH_2), 22.8 (CH_2), 22.7 (C(OBn)-*C*H_3), 14.2 (C_6H_{12}-*C*H_3); **IR** (film) 2953, 2926, 2856, 1732, 1453, 1254, 1077 cm^{-1}; **MS** (ESI+) *m/z* 346 $[M+Na]^+$; **HRMS** (ESI+) Calcd for $C_{19}H_{30}O_4Na$ $[M+Na]^+$: 345.2036, found 345.2037, and unreacted **8** (1.24 g, 30%) as a colourless oil. The overall conversion to **9** could be improved (4.73 g 82%) by deprotecting **10** to **8** as detailed below, then resubjecting the combined quantities of **8** to the benzylation conditions (two further rounds).

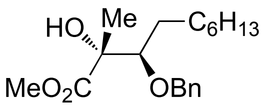

ent-**9**

Synthesis of *ent*-9, methyl (2*R*,3*R*)-3-(benzyloxy)-2-hydroxy-2-methyldecanoate. To a stirred solution of *ent*-**8** (42 mg, 0.18 mmol) in dioxane (2.00 mL) were added molecular sieves (5Å, 50.0 mg), TriBOT (24.0 mg, 0.06 mmol) and TfOH (5 mg, 0.04 mmol). After stirring for 16 h at room temperature, the reaction was quenched by addition of saturated aq. $NaHCO_3$ (5 mL). The aqueous phase was extracted with ethyl acetate (3 x 10 mL,). The combined organic phases were dried over $MgSO_4$ and concentrated *in vacuo*. The crude product was purified by column chromatography on silica (PE:EA, 19:1 to 10:1 to 5:1 to 3:1 to 2:1) to give *ent*-**9** (25 mg, 43%, mixture of isomers 5:1) as a yellow oil. : $[\alpha]_D^{32}$ +9.2 (*c* 0.4, $CHCl_3$), spectroscopic data as previously described.

Recovery of methyl (2*S*,3*S*)-2,3-dihydroxy-2-methyldecanoate, 8 from 10. A solution of **10** (1.12 g, 2.72 mmol) in EtOH (30 mL) was degassed then Pd/C (5 wt%, 100 mg) added. The reaction

mixture was stirred under an atmosphere of hydrogen (balloon) for 2 h at 35 °C. The mixture was filtered through a pad of Celite, washed with EtOH (2 x 30 mL) and concentrated *in vacuo*. The crude product was purified by column chromatography (petroleum ether: EtOAc; 3:1 →2:1) to give **8** (635 mg, quant.) as a colourless oil.

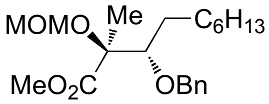

18

Methyl (2*S*,3*S*)-3-(benzyloxy)-2-(methoxymethoxy)-2-methyldecanoate, 18. Alcohol **9** (4.78 g, 14.8 mmol), DIPEA (11.5 g, 88.8 mmol) and MOMCl (3.57 g, 44.4 mmol) were dissolved in CH_2Cl_2 (45.0 mL) in a sealed tube. The reaction mixture was stirred at 40 °C for 24 h. Additional MOMCl (530 mg, 4.23 mmol) and DIPEA (1.52 g, 14.9 mmol) were added after 24 h and 48 h. On cooling, the mixture was diluted with ethyl acetate (150 mL) and quenched with aq. 2M HCl (75 mL). The aqueous phase was extracted with ethyl acetate (3 x 100 mL). The combined organic phases were washed with brine (2 x 20 mL), dried over $MgSO_4$ and concentrated *in vacuo*. The crude product was purified by column chromatography (petroleum ether:EtOAc; 20:1→10:1) to give **18** (4.98 g, 92%) as a 6:1 mixture of isomers and as a yellow oil. $[\alpha]_D^{32}$ –7.9 (*c* 0.5, $CHCl_3$); **^{1}H-NMR** (400 MHz, $CDCl_3$) δ 7.38 – 7.28 (m, 5H, ArH), 4.86 (d, *J* = 7.2 Hz, 1H, O-CH_aH$_b$-OCH$_3$), 4.64 (d, *J* = 7.2 Hz, 1H, O-CH$_a$$H_b$-OCH$_3$), 4.62 (d, *J* = 11.2 Hz, 1H, CH_aH$_b$-Ph), 4.55 (d, *J* = 11.2 Hz, 1H, CH$_a$$H_b$-Ph), 3.80 (dd, *J* = 8.8, 3.0 Hz, 1H, C*H*-OBn), 3.70 (s, 3H, CO_2CH_3), 3.39 (s, 3H, CH$_2$-O-CH_3), 1.61 – 1.52 (m, 2H, CH$_2$), 1.50 (s, 3H, C(OMOM)-CH_3), 1.35 – 1.25 (m, 10H, CH$_2$), 0.90 (t, *J* = 6.8 Hz, 3H, CH$_2$-CH_3); **^{13}C-NMR** (101 MHz, $CDCl_3$) δ 174.3 (*C*O$_2$Me), 138.7 (*C*CH$_2$O), 128.4 (*C*H(Ar-o)), 127.6 (*C*H(Ar-m)), 127.6 (*C*H(Ar-p)), 92.5 (O-CH$_2$-O), 83.9 (*C*H(OBn)), 81.6 (C(OMOM)), 74.6 (CH$_2$Bn), 56.3 (*C*H$_3$-O-CH$_2$), 52.2 (CO$_2$*C*H$_3$), 32.0 (CH$_2$), 30.7 (CH$_2$), 30.0 (CH$_2$), 29.4 (CH$_2$), 27.1 (CH$_2$), 22.8 (CH$_2$), 15.3 (C(OMOM)-*C*H$_3$), 14.2 (C$_6$H$_{12}$-*C*H$_3$); **IR** (film) 2952, 2926, 2856, 1453, 1739, 1131, 1027 cm^{-1}; **MS** (ESI+) *m/z* 389 [M+Na]$^+$; **HRMS** (ESI+) Calcd for $C_{21}H_{34}O_5Na$ [M+Na]$^+$: 389.2298, found 389.2301.

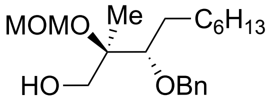

11

(2*R*,3*S*)-3-(Benzyloxy)-2-(methoxymethoxy)-2-methyldecan-1-ol, 11. To a solution of **18** (3.69 g, 10.1 mmol, 6:1 mixture of isomers) in THF (50.0 mL) was added dropwise DIBAL (1.0 M in hexane, 45.0 mL, 45.0 mmol) at 0 °C and after addition stirred for 1 h. The reaction mixture was allowed to warm to room temperature and stirred for 1 h. The reaction mixture was hydrolysed with brine (50 mL) and carefully acidified with HCl (ca. 1 M, in water, 50.0 mL). The aqueous phase was extracted with ethyl acetate (100 mL, three times). The combined organic phases were dried over $MgSO_4$ and concentrated *in vacuo*. The crude product was purified by column chromatography (petroleum ether:EtOAc; 10:1→5:1) to give **11** (2.84 g, 83%) as a pale yellow oil. $[\boldsymbol{\alpha}]_D^{32}$ 1.4 (*c* 0.4, $CHCl_3$); **^{1}H-NMR** (400 MHz, $CDCl_3$) δ 7.39 – 7.29 (m, 5H, ArH), 4.84 (d, *J* = 7.4 Hz, 1H, O-C$H_a$$H_b$-$OCH_3$), 4.75 (d, *J* = 11.2 Hz, 1H, C$H_a$$H_b$-Ph), 4.74 (d, *J* = 7.3 Hz, 1H, O-C$H_a$$H_b$-$OCH_3$), 4.65 (d, *J* = 11.2 Hz, 1H, C$H_a$$H_b$-Ph), 3.73 (d, *J* = 12.4 Hz, 1H, C$H_a$$H_b$OH), 3.62 (d, *J* = 12.4 Hz, 1H, C$H_a$$H_b$OH), 3.58 (dd, *J* = 9.4, 2.1 Hz, 1H, C*H*-OBn), 3.46 (s, 3H, OCH_3), 3.28 (s, 1H, OH), 1.64 – 1.52 (m, 2H, CH_2), 1.37 – 1.23 (m, 10H, CH_2), 1.16 (s, 3H, C(OMOM)-CH_3), 0.91 (t, *J* = 6.8 Hz, 3H, CH_2-CH_3); **^{13}C-NMR** (101 MHz, $CDCl_3$) δ 139.0 (*C*CH_2O), 128.5 (*C*H(Ar-o)), 127.8 (*C*H(Ar-m)), 127.7 (*C*H(Ar-p)), 91.1 (O-CH_2-O), 82.0 (C(OMOM)), 81.8 (*C*H(OBn)), 74.9 (CH_2Bn), 65.7 (CH_2OH), 55.7 (*C*H_3-O-CH_2), 32.0 (CH_2), 30.6 (CH_2), 30.1 (CH_2), 29.4 (CH_2), 27.3 (CH_2), 22.8 (CH_2), 15.9 (C(OMOM)-*C*H_3), 14.3 (C_6H_{12}-*C*H_3); **IR** (film) 2924, 2855, 1722, 1496, 1094, 1027 cm^{-1}; **MS** (ESI+) *m/z* 361 $[M+Na]^+$; **HRMS** (ESI+) Calcd for $C_{20}H_{34}O_4Na$ $[M+Na]^+$: 361.2349, found 361.2350.

(*R*)-5-((*S*)-1-(Benzyloxy)octyl)-8,8-diisopropyl-5,9-dimethyl-2,4,7-trioxa-8-siladecane, 19. To a solution of **11** (1.01 g, 3.00 mmol) in CH_2Cl_2 (30 mL) were added 2,6-lutidine (642 mg, 6.00 mmol) and a solution of TIPSOTf (1.38 g, 4.50 mmol) in CH_2Cl_2 (5 mL) at 0 °C. The reaction mixture was stirred at 0 °C for 1 h and quenched by addition of brine (20 mL) and aq. 1 M HCl (2 mL). The aqueous phase was extracted with ethyl acetate (3 x 100 mL). The combined organic phases were dried over $MgSO_4$ and concentrated *in vacuo*. The crude product was purified by column chromatography (petroleum ether:EtOAc; 20:1) to give **19** (1.48 g, quant.) as a pale yellow oil. $[\boldsymbol{\alpha}]_{\boldsymbol{D}}^{\mathbf{32}}$ –6.5 (*c* 0.1, $CHCl_3$); **^{1}H-NMR** (400 MHz, $CDCl_3$) δ 7.37 – 7.26 (m, 5H, ArH), 4.91 (d, *J* = 6.9 Hz, 1H, O-$CH_a\mathit{H_b}$-OCH_3), 4.86 (d, *J* = 6.9 Hz, 1H, O-$C\mathit{H_a}H_b$-OCH_3), 4.64 (s, 2H, $C\mathit{H_2}Ph$), 3.78 (d, *J* = 10.1 Hz, 1H, $C\mathit{H_a}H_bOTIPS$), 3.73 (d, *J* = 10.1 Hz, 1H, $CH_a\mathit{H_b}OTIPS$), 3.53 (dd, *J* = 9.0, 2.6 Hz, 1H, C*H*-OBn), 3.38 (s, 3H, OCH_3), 1.79 – 1.66 (m, 1H, $C\mathit{H_a}H_b$), 1.65 – 1.51 (m, 3H, CH_2 + $CH_a\mathit{H_b}$), 1.36 – 1.26 (m, 8H, CH_2),1.34 (s, 3H, C(OMOM)-$C\mathit{H_3}$), 1.11 – 1.06 (m, 21H, $Si(C\mathit{H}(C\mathit{H_3})_2)_3$), 0.90 (t, *J* = 6.8 Hz, 3H, C_6H_{12}-$C\mathit{H_3}$); **^{13}C-NMR** (101 MHz, $CDCl_3$) δ 139.2 (*C*CH_2O), 128.4 (*C*H(Ar-o)), 127.7 (*C*H(Ar-m)), 127.5 (*C*H(Ar-p)), 92.1 (O-CH_2-O), 83.0 (*C*H(OBn)), 81.4 (C(OMOM)), 74.7 (CH_2Bn), 67.9 (CH_2OTIPS), 55.5 ($\mathit{C}H_3$-O-CH_2), 32.0 (CH_2), 30.6 (CH_2), 30.2 (CH_2), 29.5 (CH_2), 27.3 (CH_2), 22.8 (CH_2), 18.2 (3x CH_3, $SiCH(\mathit{C_a}H_3)(C_bH_3)$), 18.2 (3x CH_3, $SiCH(C_aH_3)(\mathit{C_b}H_3)$), 17.1 (C(OMOM)-$\mathit{C}H_3$), 14.3 ($C_6H_{12}$-$\mathit{C}H_3$), 12.1 (3x CH, SiCH); **IR** (film) 2925, 2865, 1724, 1463, 1098, 1029 cm^{-1}; **MS** (ESI+) *m/z* 517 $[M+Na]^+$; **HRMS** (ESI+) Calcd for $C_{29}H_{54}O_4SiNa$ $[M+Na]^+$: 517.3684, found 517.3685.

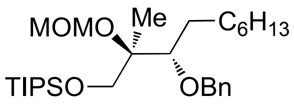

19

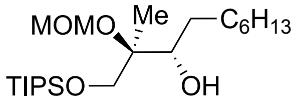

12

(2*R*,3*S*)-2-(Methoxymethoxy)-2-methyl-1-((triisopropylsilyl)oxy)decan-3-ol, 12. A solution of **19** (1.47 g, 2.97 mmol) in EtOH (50 mL) was degassed and Pd/C (5 wt%, 150 mg) added. The reaction mixture was stirred under an atmosphere of hydrogen (balloon) for 2 h at 35 °C. The reaction mixture was filtrated though a pad of celite, washed with EtOH (2 x 50 mL) and concentrated *in vacuo*. The crude product was purified by column chromatography (petroleum ether:EtOAc; 20:1→10:1) to give **12** (1.20 g, quant.) as a colourless oil. $[\alpha]_D^{32}$ –19.5 (*c* 0.1, $CHCl_3$); **^{1}H-NMR** (400 MHz, $CDCl_3$) δ 4.79 (d, *J* = 7.3 Hz, 1H, O-CH_aH_b-OCH_3), 4.73 (d, *J* = 7.3 Hz, 1H, O-CH_aH_b-OCH_3), 3.76 (d, *J* = 13.0 Hz, 1H, CH_aH_bOTIPS), 3.74 (d, *J* = 13.1 Hz, 1H, CH_aH_bOTIPS), 3.64 – 3.55 (m, 1H, *CH*OH), 3.37 (s, 3H, OCH_3), 3.11 (d, *J* = 4.8 Hz, 1H, OH), 1.63 – 1.49 (m, 2H, CH_2), 1.34 – 1.21 (m, 13H, CH_2 + CH_3), 1.11 – 1.01 (m, 21H, $Si(CH(CH_3)_2)_3$), 0.87 (t, *J* = 6.7 Hz, 3H, C_6H_{12}-CH_3); **^{13}C-NMR** (101 MHz, $CDCl_3$) δ 91.6 (O-CH_2-O), 80.8 (C(OMOM)), 76.1 (COH), 68.6 (CH_2OTIPS), 55.6 (CH_3-O-CH_2), 32.0 (CH_2), 31.3 (CH_2), 29.9 (CH_2), 29.5 (CH_2), 26.9 (CH_2), 22.8 (CH_2), 18.1 (6x CH_3, $SiCH(CH_3)_2$), 17.1 (C(OMOM)-CH_3), 14.2 (C_6H_{12}-CH_3), 12.0 (3x CH, SiCH); **IR** (film) 2923, 2866, 1463, 1260, 1215, 1033 cm^{-1}; **MS** (ESI+) *m/z* 427 $[M+Na]^+$; **HRMS** (ESI+) Calcd for $C_{22}H_{48}O_4SiNa$ $[M+Na]^+$: 427.3214, found 427.3217.

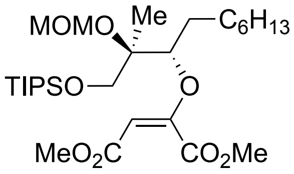

13

Dimethyl 2-(((2*R*,3*S*)-2-(methoxymethoxy)-2-methyl-1-((triisopropyl-silyl)oxy)-decan3-yl)oxy)-maleate, 13. To a solution of **12** (740 mg, 1.83 mmol) in THF (36 mL) at –78 °C was added *n*-BuLi (2.0 M in *n*-hexane, 20 mol%, 0.18 mL). The reaction mixture was stirred for 15 minutes at this temperature, then a solution of dimethyl acetylenedicarboxylate (freshly destilled, 520 mg, 3.66 mmol) in THF (10.0 mL) was added dropwise via syringe pump over 40 minutes. The reaction mixture was allowed to warm to 0 °C over 4 h. The reaction was quenched by addition of saturated NH_4Cl solution and brine (1:1, 5 mL). The aqueous phase was extracted with ethyl acetate (3 x 100 mL). The combined organic phases were dried over $MgSO_4$ and concentrated *in vacuo*. The crude product was purified by column chromatography (petroleum ether:EtOAc; 20:1→13:1) to give **13** (741 mg, 74%) as a pale yellow oil. $[\boldsymbol{\alpha}]_{\boldsymbol{D}}^{\mathbf{32}}$ +4.9 (*c* 0.2, $CHCl_3$); **^{1}H-NMR** (400 MHz, $CDCl_3$) δ 5.25 (s, 1H, C=CH), 4.81 (d, *J* = 7.1 Hz, 1H, O-CH_aH_b-OCH_3), 4.72 (d, *J* = 7.1 Hz, 1H, O-CH_aH_b-OCH_3), 4.37 (dd, *J* = 8.8, 3.3 Hz, 1H, CHOC=C), 3.87 (s, 3H, $C_bO_2CH_3$), 3.70 (d, *J* = 10.2 Hz, 1H, CH_aH_bOTIPS), 3.67 (s, 3H, $C_aO_2CH_3$), 3.62 (d, *J* = 10.1 Hz, 1H, CH_aH_bOTIPS), 3.34 (s, 3H, OCH_3), 1.79 – 1.67 (m, 2H, CH_2), 1.54 – 1.41 (m, 1H, CH_aH_b), 1.32 (s, 3H, CH_3), 1.30 – 1.22 (m, 9H, CH_aH_b+ CH_2), 1.11 – 1.00 (m, 21H, $Si(CH(CH_3)_2)_3$), 0.87 (t, *J* = 6.8 Hz, 3H, C_6H_{12}-CH_3); **^{13}C-NMR** (101 MHz, $CDCl_3$) δ 167.0 (C_aO_2Me), 164.3 (C_bO_2Me), 163.1 (*C*=CH), 93.4 (C=*C*H), 91.9 (O-CH_2-O), 83.6 (*C*HOC=C), 80.2 (C(OMOM)), 67.4 (CH_2OTIPS), 55.6 (CH_3-O-CH_2), 52.9 ($C_bO_2CH_3$), 51.6 ($C_aO_2CH_3$), 31.9 (CH_2), 29.9 (CH_2), 29.4 (CH_2), 29.2 (CH_2), 26.3 (CH_2), 22.7 (CH_2), 18.1 ((3 x CH_3, $SiCH(C_aH_3)(C_bH_3)$)), 18.1 (3 x CH_3, $SiCH(C_aH_3)(C_bH_3)$)), 17.3 (C(OMOM)-CH_3), 14.2 (C_6H_{12}-CH_3), 12.0 (3 x CH, SiCH); **IR** (film) 2927, 2866, 1755, 1723, 1623, 1462, 1437, 1366, 1141 cm^{-1}; **MS** (ESI+) *m/z* 569 $[M+Na]^+$; **HRMS** (ESI+) Calcd for $C_{28}H_{54}O_8SiNa$ $[M+Na]^+$: 569.3480, found 569.3485.

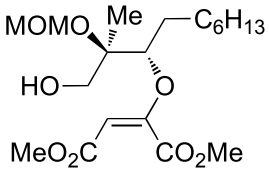

20

Dimethyl 2-(((2*R*,3*S*)-1-hydroxy-2-(methoxymethoxy)-2-methyldecan-3-yl)oxy)maleate, 20. To a solution of **13** (820 mg, 1.50 mmol) in THF (5 mL) in a polyethylene-vial was added a solution of HF·Pyridine (2.5 mL) in pyridine (5.0 ml) (CAUTION). The reaction mixture was stirred for 18 h at 35 °C until complete consumption of the starting material (by TLC). The mixture was carefully quenched via the dropwise addition to a solution of $NaHCO_3$ (200 mL). The aqueous phase was extracted with ethyl acetate (3 x 100 mL). The combined organic phases were dried over $MgSO_4$ and concentrated *in vacuo*. The crude product was purified by column chromatography (petroleum ether:EtOAc; 3:2→1:1) to give **20** (538 mg, 92%) as a colourless oil. $[\boldsymbol{\alpha}]_D^{32}$ +4.1 (*c* 0.1, $CHCl_3$); **^{1}H-NMR** (400 MHz, $CDCl_3$) δ 5.41 (s, 1H, C=CH), 4.77 (d, *J* = 7.5 Hz, 1H, O-CH_a*H_b*-OCH_3), 4.56 (d, *J* = 7.5 Hz, 1H, O-C*H_a*H_b-OCH_3), 4.27 (dd, *J* = 9.6, 2.6 Hz, 1H, CHOC=C), 3.81 (s, 3H, $C_bO_2CH_3$), 3.62 (s, 3H, $C_aO_2CH_3$), 3.46 (s, 2H, C*H_2*OH), 3.36 (s, 3H, OCH_3), 2.93 (s, 1H, OH), 1.74 – 1.62 (m, 1H, C*H_a*H_b), 1.59 – 1.47 (m, 1H, CH_a*H_b*), 1.45 – 1.35 (m, 1H, C'*H_a*H_b) 1.26 – 1.16 (m, 9H, C'H_a*H_b*, CH_2), 1.08 (s, 3H, CH_3), 0.81 (t, *J* = 6.7 Hz, 3H, C_6H_{12}-C*H_3*); **^{13}C-NMR** (101 MHz, $CDCl_3$) δ 166.9 (*C_a*O_2Me), 164.3 (*C_b*O_2Me), 162.3 (*C*=CH), 94.0 (C=*C*H), 90.9 (O-CH_2-O), 81.3 (*C*HOC=C), 80.8 (C(OMOM)), 64.5 (CH_2OH), 55.8 (*C*H_3-O-CH_2), 52.9 (C_bO_2*C*H_3), 51.5 (C_aO_2*C*H_3), 31.8 (CH_2), 29.7 (CH_2), 29.3 (CH_2), 29.1 (CH_2), 26.2 (CH_2), 22.6 (CH_2), 15.1 (C(OMOM)-*C*H_3), 14.1 (C_6H_{12}-*C*H_3); **IR** (film) 2953, 2924, 2854, 1753, 1720, 1624, 1370, 1172, 1035 cm^{-1}; **MS** (ESI+) *m/z* 413 $[M+Na]^+$; **HRMS** (ESI+) Calcd for $C_{19}H_{34}O_8Na$ $[M+Na]^+$: 413.2146, found 413.2147.

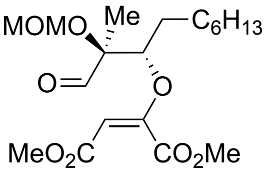

14

Dimethyl 2-(((2*S*,3*S*)-2-(methoxymethoxy)-2-methyl-1-oxodecan-3-yl)oxy)maleate, 14. To a solution of **20** (298 mg, 0.76 mmol) in CH_2Cl_2 (10 mL) were added powdered 4Å molecular sieves (2.5 g), *N*-methylmorpholine *N*-oxide (148 mg, 0.84 mmol) and tetrapropylammonium perruthenate (13.4 mg, 5 mol%). The reaction mixture was stirred for 0.5 h at room temperature then the whole mixture, without concentration, was purified by column chromatography (petroleum ether:EtOAc; 5:1→3:1) to give **14** (267 mg, 90%) as a colourless oil. $[\alpha]_D^{32}$ –10.6 (*c* 0.3, $CHCl_3$); **^{1}H-NMR** (400 MHz, $CDCl_3$) δ ; 9.60 (s, 1H, CH(=O)), 5.34 (s, 1H, C=CH), 4.85 (d, *J* = 7.3 Hz, 1H, O-CH_aH_b-OCH_3), 4.64 (d, *J* = 7.3 Hz, 1H, O-CH_aH_b-OCH_3), 4.32 (dd, *J* = 8.8, 3.9 Hz, 1H, CHOC=C), 3.87 (s, 3H, $C_bO_2CH_3$), 3.69 (s, 3H, $C_aO_2CH_3$), 3.40 (s, 3H, OCH_3), 1.68 – 1.55 (m, 2H, CH_2), 1.46 – 1.39 (m, 1H, C'H_aH_b), 1.35 (s, 3H, CH_3), 1.31 – 1.21 (m, 9H, C'H_aH_b, CH_2), 0.87 (t, *J* = 6.8 Hz, 3H, C_6H_{12}-CH_3). **^{13}C-NMR** (101 MHz, $CDCl_3$) δ 202.1 (C=O), 166.6 (C_aO_2Me), 163.9 (C_bO_2Me), 162.2 (*C*=CH), 94.7 (C=*C*H), 92.1 (O-CH_2-O), 83.9 (C(OMOM)), 83.5 (*C*HOC=C), 56.1 (CH_3-O-CH_2), 53.0 ($C_bO_2CH_3$), 51.8 ($C_aO_2CH_3$), 31.8 (CH_2), 29.9 (CH_2), 29.6 (CH_2), 29.1 (CH_2), 25.9 (CH_2), 22.7 (CH_2), 14.8 (C(OMOM)-CH_3), 14.2 (C_6H_{12}-CH_3); **IR** (film) 2954, 2857, 1750, 1722, 1627, 1438, 1449 cm^{-1}; **MS** (ESI+) *m/z* 411 $[M+Na]^+$; **HRMS** (ESI+) Calcd for $C_{19}H_{32}O_8Na$ $[M+Na]^+$: 411.1989, found 411.1993.

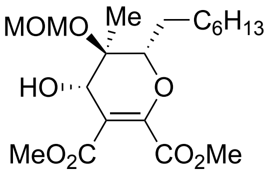

15

Dimethyl (2*S*,3*R*,4*S*)-2-heptyl-4-hydroxy-3-(methoxymethoxy)-3-methyl-3,4-dihydro-2*H*-pyran-5,6-dicarboxylate, 15. A solution of **14** (300 mg, 0.77 mmol) in THF (3.0 mL) was added to NaHMDS (1.0 M in THF, 1.16 mL, 1.16 mmol) in THF (10.0 mL) at –78 °C. After stirring for 0.5 h at –78 °C, the reaction was quenched by addition of saturated NH_4Cl and brine (1:2, 21 mL). The aqueous phase was extracted with ethyl acetate (3 x 50 mL). The combined organic phases were dried over $MgSO_4$ and concentrated *in vacuo*. The crude product was purified by column chromatography (petroleum ether:EtOAc; 5:1→3:1) to give **15** (110 mg, 37%) as a colourless oil. $[\alpha]_D^{32}$ –32.6 (*c* 0.20, $CHCl_3$); **^{1}H-NMR** (400 MHz, $CDCl_3$) δ 4.89 (d, *J* = 7.6 Hz, 1H, O-CH_aH_b-OCH_3), 4.78 (d, *J* = 7.6 Hz, 1H, O-CH_aH_b-OCH_3), 4.54 (d, *J* = 2.7 Hz, 1H, C*H*OH), 3.97 (dd, *J* = 8.9, 2.8 Hz, 1H, CHOC=C), 3.84 (s, 3H, $C_bO_2CH_3$), 3.77 (s, 3H, $C_aO_2CH_3$), 3.65 (s, br, 1H, CHO*H*), 3.41 (s, 3H, OCH_3), 1.78 – 1.67 (m, 2H, CH_2), 1.60 – 1.53 (m, 1H, C'H_aH_b), 1.36 – 1.23 (m, 9H, C'H_aH_b, CH_2), 1.30 (s, 3H, CH_3), 0.88 (t, *J* = 6.6 Hz, 3H, C_6H_{12}-CH_3); **^{13}C-NMR** (101 MHz, $CDCl_3$) δ 166.7 ($C_aO_2CH_3$), 163.3 ($C_bO_2CH_3$), 151.3 (O*C*=*C*), 109.3 (OC=*C*), 91.5 (O-CH_2-O), 82.8 (*C*HOC=C), 75.8 (C(OMOM)), 70.1 (COH), 56.1 (*C*H_3-O-CH_2), 52.9 ($C_bO_2CH_3$), 52.3 ($C_aO_2CH_3$), 31.9 (CH_2), 29.5 (CH_2), 29.3 (CH_2), 27.6 (CH_2), 26.6 (CH_2), 22.7 (CH_2), 14.2 (C_6H_{12}-CH_3), 12.2 (C(OMOM)-CH_3); **IR** (film) 2956, 1750, 1620, 1450, 1301 cm^{-1}; **MS** (ESI+) *m/z* 411 $[M+Na]^+$; **HRMS** (ESI+) Calcd for $C_{19}H_{32}O_8Na$ $[M+Na]^+$: 411.1989, found 411.1993.

(2*S*,3*R*,4*S*)-2-heptyl-4-hydroxy-6-methoxy-3-(methoxymethoxy)-3-methyl-3,4-dihydropyrano-[2,3-c]pyrrole-5,7(2H,6H)-dione, 17.

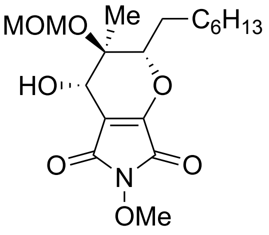

17

To a stirred solution of **15** (140 mg, 0.36 mmol) in MeOH (5 mL) was added aq. 1 M NaOH (0.8 mL). The reaction mixture was stirred overnight at 35 °C then acidified by addition of aq. 1M HCl (2 mL). The crude mixture was extracted with ethyl acetate (3 x 30 mL), the combined organic phases dried over Na_2SO_4 and concentrated *in vacuo*. This crude acid was used without further purification. To a solution of the acid (123 mg) in CH_2Cl_2 (15 mL) were added $MeONH_2$•HCl (75.2 mg, 0.90 mmol), NEt_3 (145mg, 1.44 mmol), HOBt•H_2O (97.2 mg, 0.72 mmol) and EDC•HCl (138 mg, 0.72 mmol). The reaction mixture was stirred over night at room temperature and after concentration *in vacuo* purified by column chromatography (petroleum ether:EtOAc; 2:1→1:2) to give **17** (24.1 mg, 18%) as a yellow oil and **16** (58 mg, 32%) as a yellow oil. To a solution of **16** (58 mg, 0.11 mmol) in DMF (2.0 mL) was added NEt_3 (0.1 mL) and stirred at 60°C for 4 h. The crude mixture was purified after concentration *in vacuo* by column chromatography (petroleum ether:EtOAc; 1:1) to give **17** (31 mg, 73%) as a colourless oil. $[\alpha]_D^{32}$ –40.5 (*c* 0.3, $CHCl_3$); **^{1}H-NMR** (400 MHz, $CDCl_3$) δ 4.93 (d, *J* = 7.7 Hz, 1H, O-CH_aH_b-OCH_3), 4.65 (d, *J* = 7.8 Hz, 1H, O-CH_aH_b-OCH_3), 4.56 (s, 1H, C*H*OH), 4.11 (d, *J* = 8.9 Hz, 1H, CHOC=C), 3.95 (s, 3H, $NOCH_3$), 3.43 (s, 3H, OCH_3), 2.16 (s, 1H, OH), 1.88 – 1.60 (m, 3H, CH_2, C'H_aH_b), 1.33 – 1.20 (m, 9H, CH_2, C'H_aH_b), 1.26 (s, 3H, CH_3), 0.81 (d, *J* = 6.9 Hz, 3H, C_6H_{12}-CH_3); **^{13}C-NMR** (101 MHz, $CDCl_3$) δ 164.7 (C_a=O), 160.5 (C_b=O), 152.9 (O*C*=*C*), 109.4 (OC=*C*), 91.3 (O-CH_2-O), 86.0 (*C*HOC=C), 77.1 (C(OMOM)), 68.5 (COH), 66.2 ($NOCH_3$), 56.3 (*C*H_3-O-CH_2), 31.9 (CH_2), 29.5 (CH_2), 29.2 (CH_2), 27.9 (CH_2), 26.5 (CH_2), 22.7 (CH_2), 14.2 (C_6H_{12}-*C*H_3), 11.1 (C(OMOM)-*C*H_3); **IR** (film) 3410, 2935, 1672, 1560, 1457, 1225, 1050 cm^{-1}; **MS** (ESI+) *m/z* 394 $[M+Na]^+$; **HRMS** (ESI+) Calcd for $C_{18}H_{29}NO_7Na$ $[M+Na]^+$: 394.1836, found 394.1839.

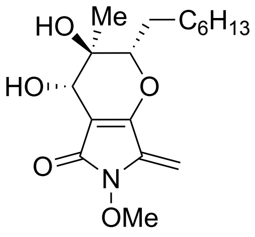

Phyllostictine A

(2*S*,3*R*,4*S*)-2-Heptyl-3,4-dihydroxy-6-methoxy-3-methyl-7-methylene-3,4,6,7-tetrahydropyrano[2,3-c]pyrrol-5(2*H*)-one (*phyllostictine A*). To **17** (8.1 mg, 0.022 mmol) in Et_2O (2.0 mL) at –78°C was added MeMgBr (3.0 M in Et_2O, 0.15 mL, 0.45 mmol) dropwise. The reaction mixture was stirred for 25 min until complete consumption of the starting material then quenched with saturated NH_4Cl solution (5.0 mL) and allowed to warm to room temperature. The aqueous phase was extracted with ethyl acetate (3 x 20 mL), the organic phases combined and dried over Na_2SO_4 then concentrated *in vacuo*. This material was used without further purification. The crude alcohol (8 mg) was dissolved in 10% TFA in CH_2Cl_2 (2 mL) and stirred at room temperature for 30 min. The mixture was concentrated *in vacuo* and crude material purified by preparative TLC on silica (petroleum ether:EtOAc; 1:2) followed by preparative reverse phase HPLC to give phyllostictine A (2.5 mg, 35%) as a colourless oil. The HPLC purification was conducted on an Agilent PLRP-S RP 100Å (150 x 25 mm, 8 µm) column. The mobile phase consisted of a gradient of water and acetonitrile (HPLC grade) at a flow rate of 10 mL/min, with UV detection at 210, 254 and 280 nm. Elution Gradient: 0-3 min 5% AcCN; 3-10 min, 5-75% AcCN; 10-30 min, 75-100% AcCN; R_t: 16.21 min. $[\alpha]_D^{21}$ –83.3 (*c* 0.03, $CHCl_3$); **^{1}H-NMR** (500 MHz, DMSO-d_6) δ 5.37 (s, 1H, OH), 4.96 (s, 1H, C=CH_aH$_b$), 4.96 (s, 1H, C=CH$_a$$H_b$), 4.85 (s, 1H, OH), 4.00 (d, *J* = 11.3 Hz, 1H, CHOC=C), 3.79 (s, 1H, C*H*OH), 3.72 (s, 3H, OCH_3), 1.87 – 1.77 (m, 1H, C'H_aH$_b$), 1.56 – 1.31 (m, 2H, C'H$_a$$H_b$, C'' H_aH$_b$), 1.25 – 1.14 (m, 9H, CH_2, C''H$_a$$H_b$), 1.11 (s, 3H, CH_3), 0.79 (t, *J* = 6.7 Hz, 3H, C_6H_{12}-CH_3); **^{13}C-NMR** (125 MHz, DMSO-d_6) δ 166.5 (C=O), 155.3 (O*C*=*C*), 137.1 (N*C*=C), 104.8 (OC=*C*), 90.9 (NC=*C*H_2), 86.3 (*C*HOC=C), 71.0 (*C*(OH)CH_3), 64.3 (CHOH), 63.8 ($NOCH_3$), 31.2 (CH_2), 28.7 (CH_2), 28.6 (CH_2), 27.6 (CH_2), 26.5 (CH_2), 22.1 (CH_2), 19.9 (CH_3) 14.0 (C_6H_{12}-*C*H_3); **IR** (film) 3389, 2926, 1707, 1635, 1453, 1379, 1065 cm^{-1}; **MS** (ESI+) *m/z* 348 $[M+Na]^+$; **HRMS** (ESI+) Calcd for $C_{17}H_{27}NO_5Na$ $[M+Na]^+$: 348.1781, found 348.1781.

3. Copies of ^{1}H and ^{13}C NMR spectra

^{1}H-NMR (400 MHz, $CDCl_3$)

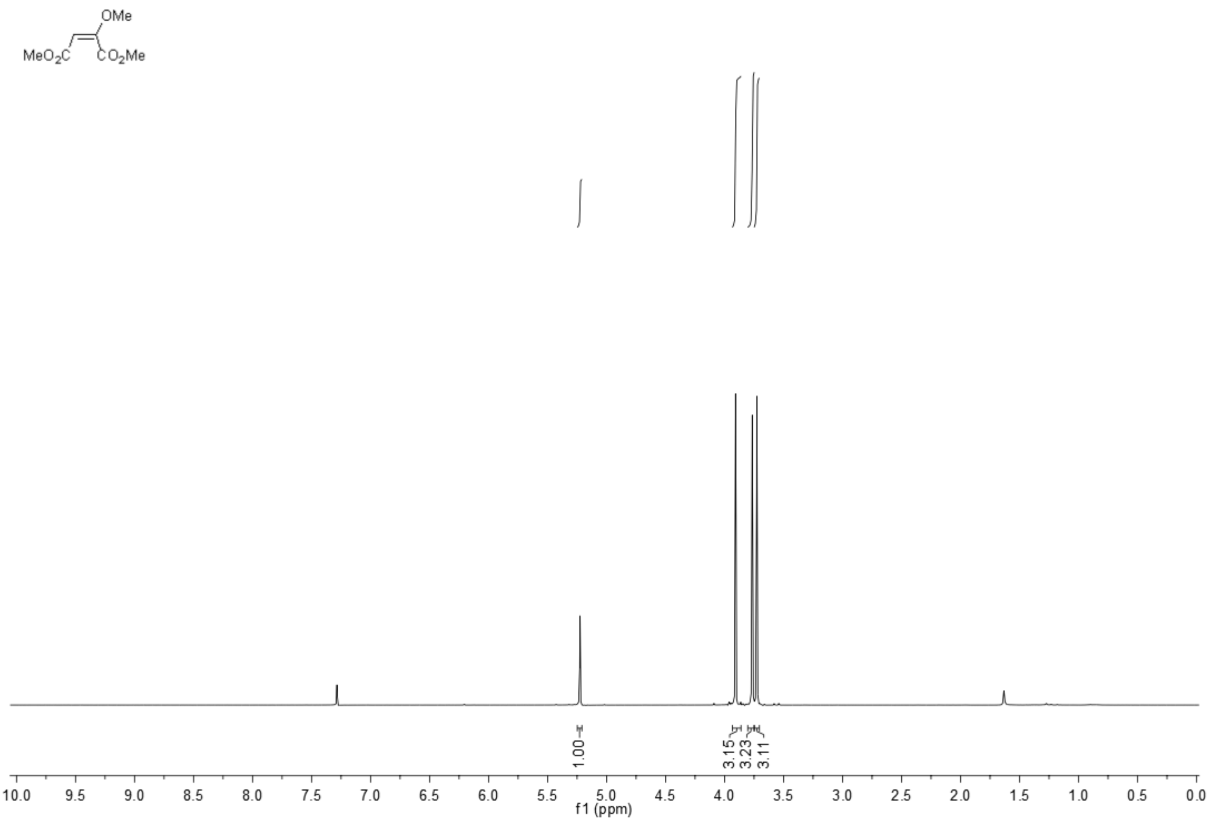

^{13}C-NMR (101 MHz, $CDCl_3$)

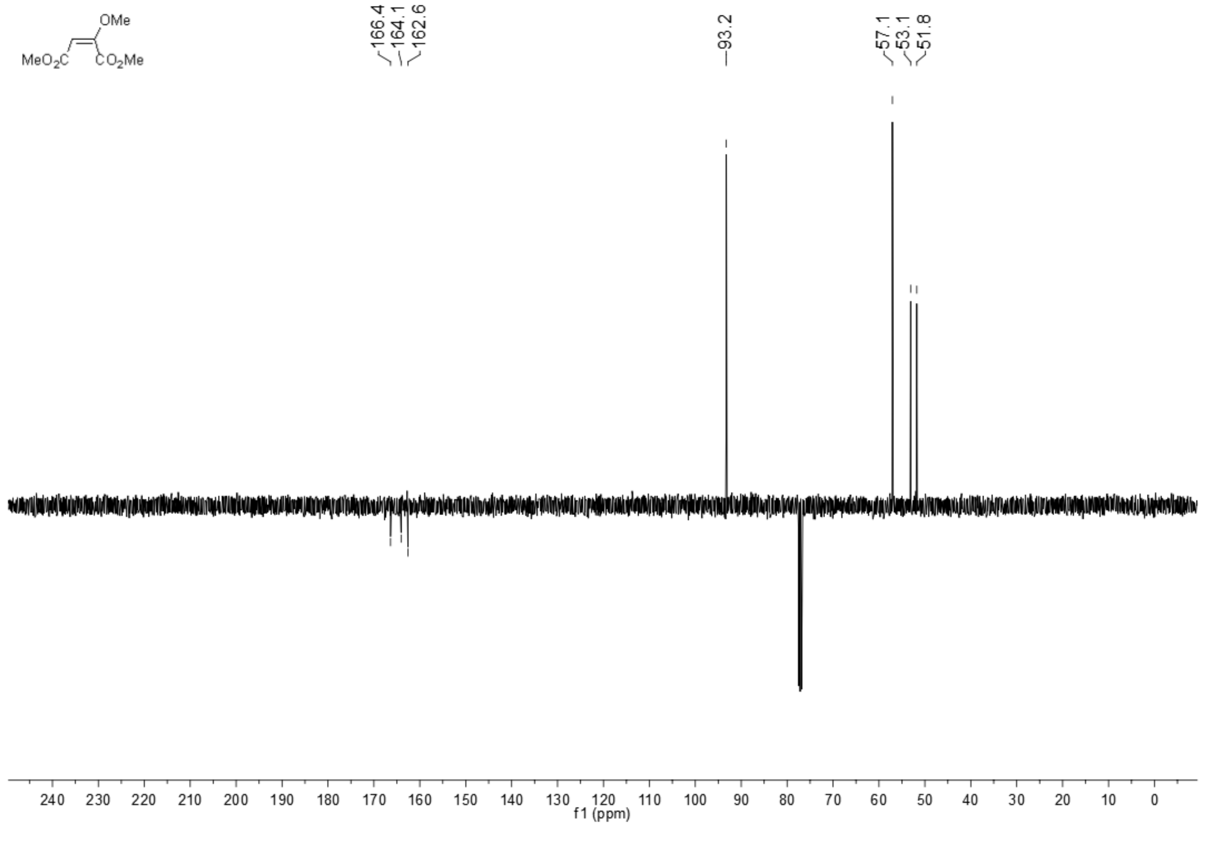

^{1}H-NMR (400 MHz, $CDCl_3$)

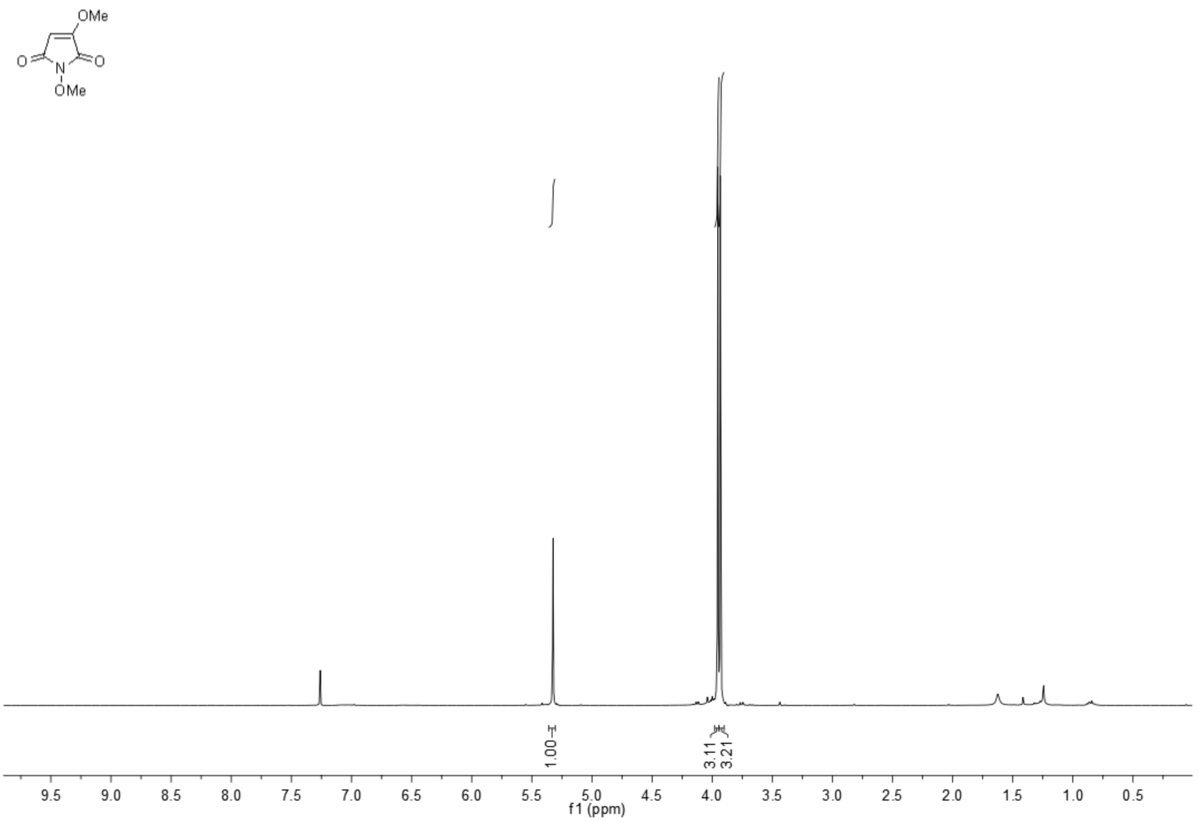

^{13}C-NMR (101 MHz, $CDCl_3$)

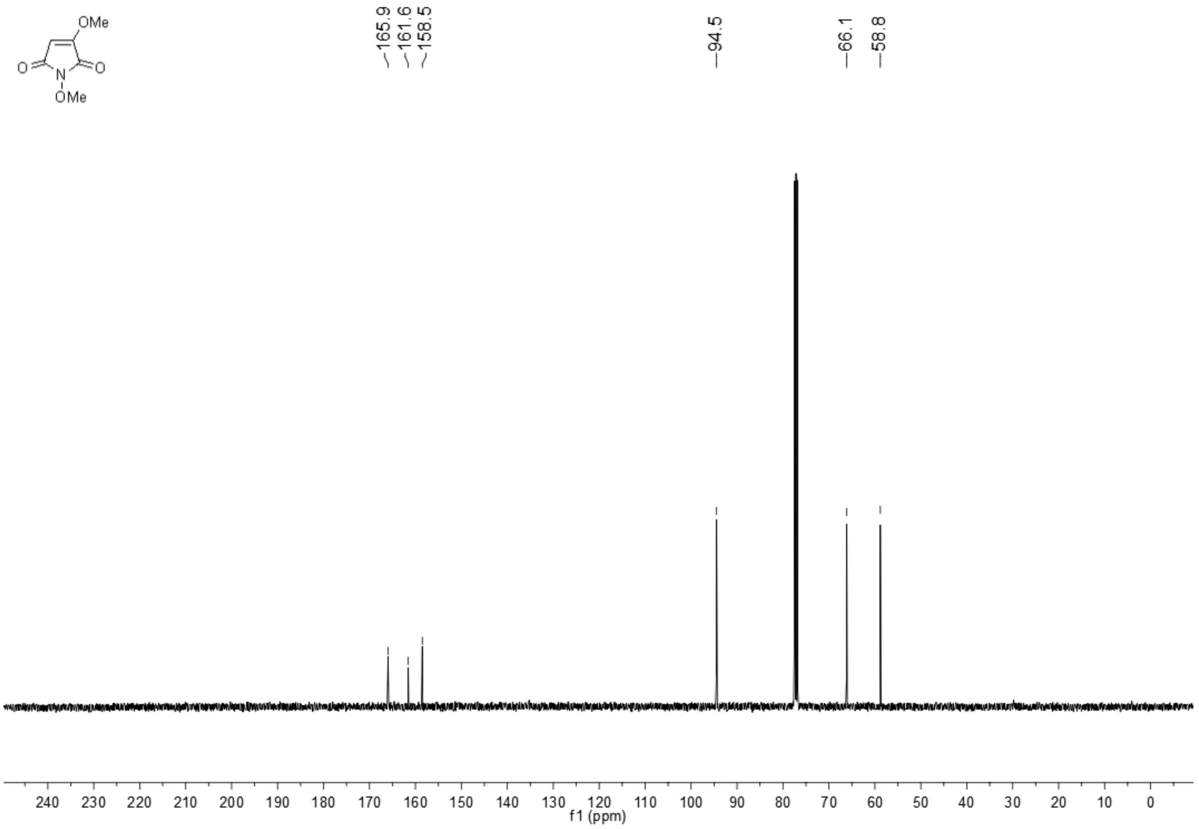

^{1}H-NMR (400 MHz, acetone-d_6)

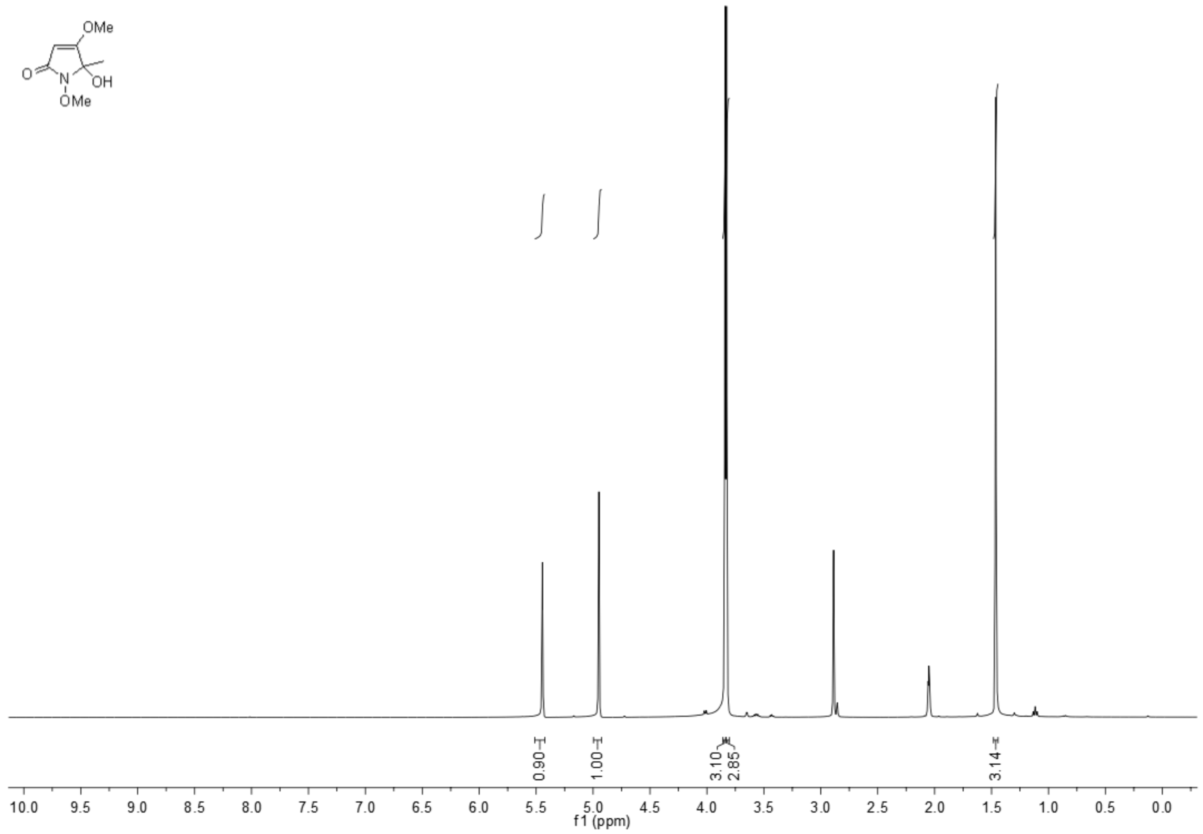

^{13}C-NMR (101 MHz, acetone-d_6

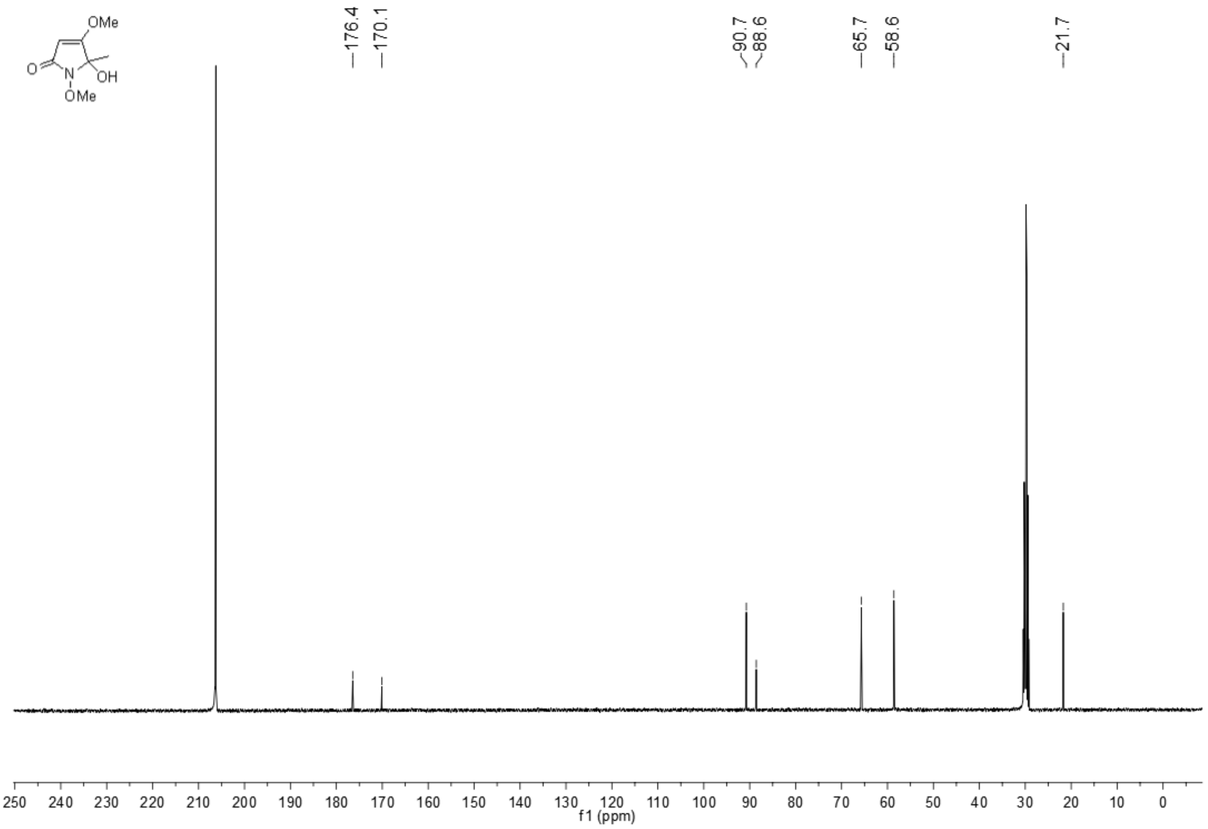

)

^{1}H-NMR (400 MHz, acetone-d$_6$)

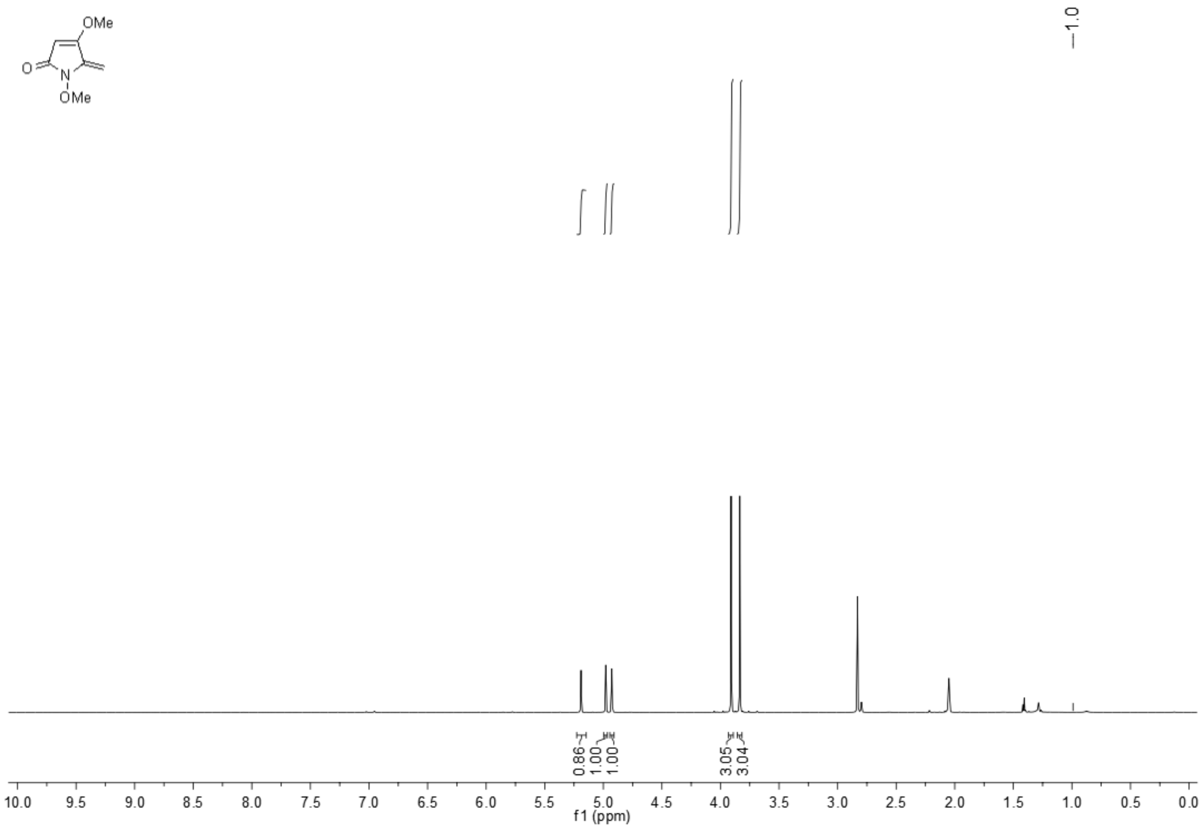

^{13}C-NMR (101 MHz, acetone-d$_6$)

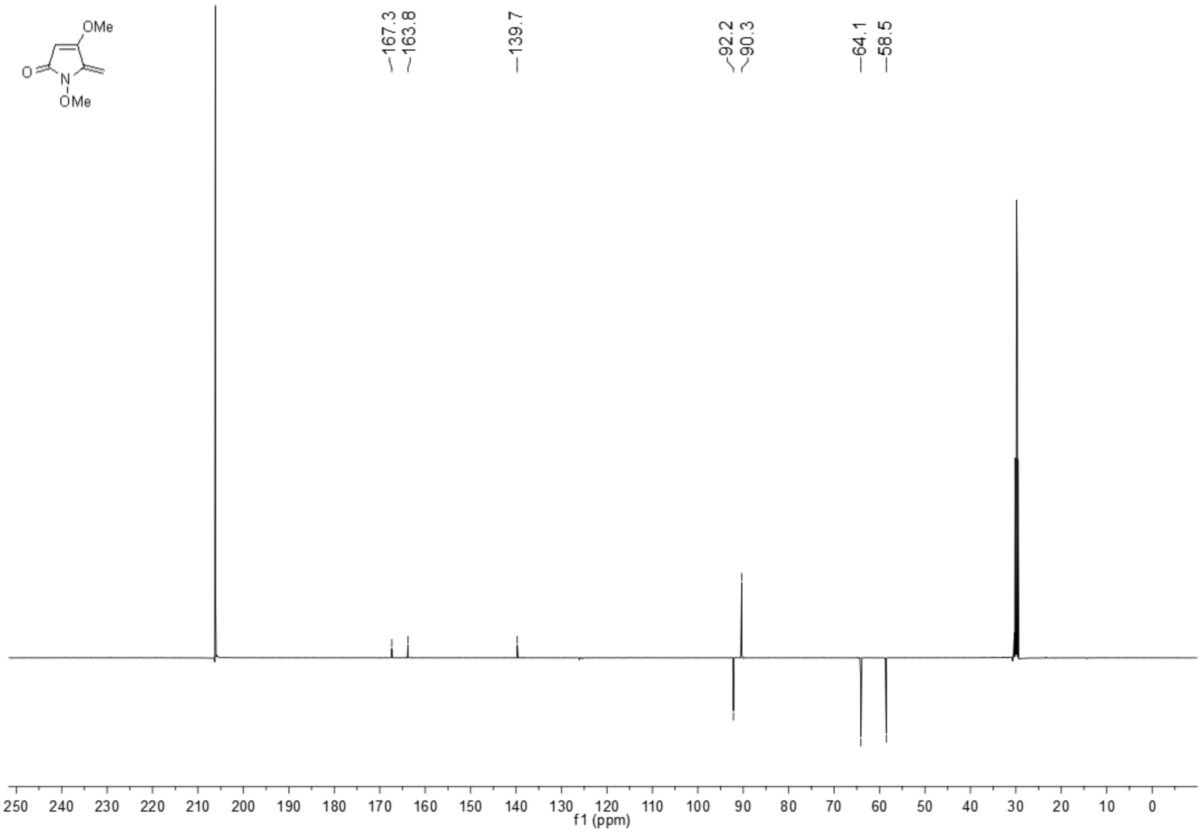

^{1}H-NMR (400 MHz, $CDCl_3$)

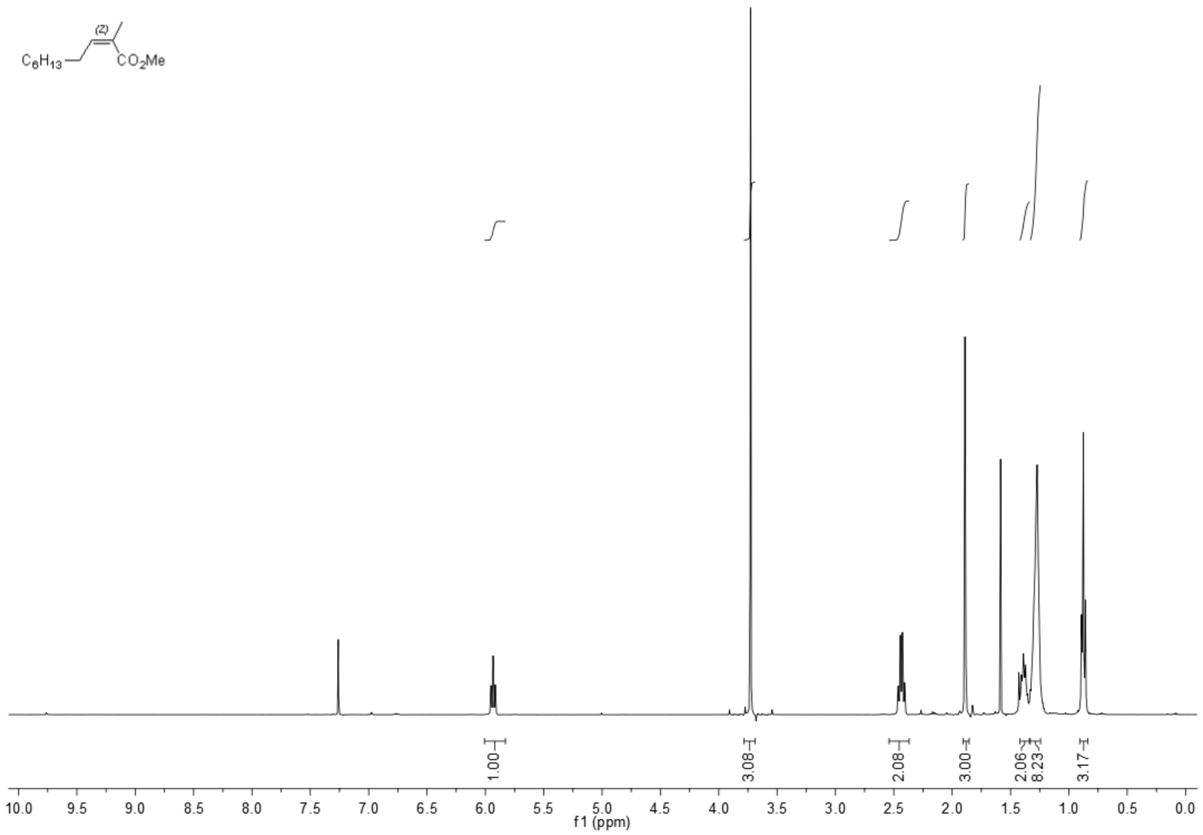

^{13}C-NMR (101 MHz, $CDCl_3$)

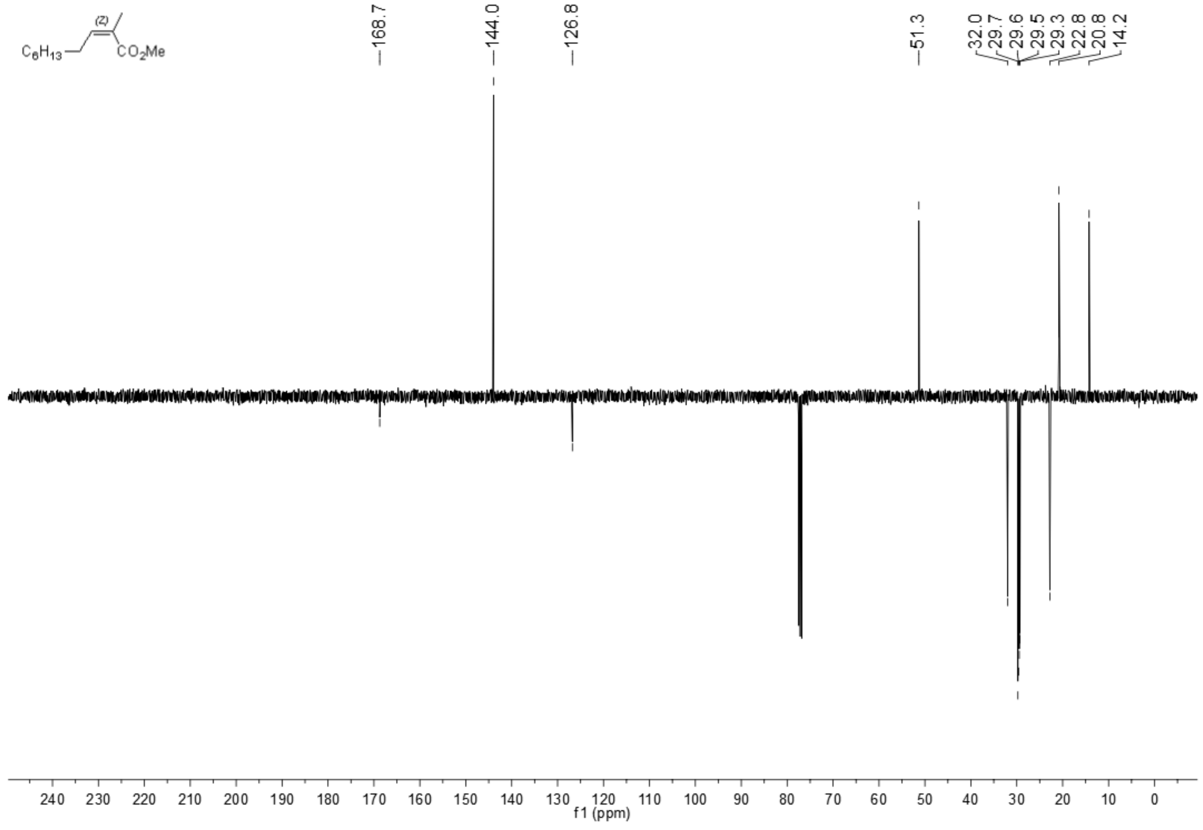

^{1}H-NMR (400 MHz, $CDCl_3$)

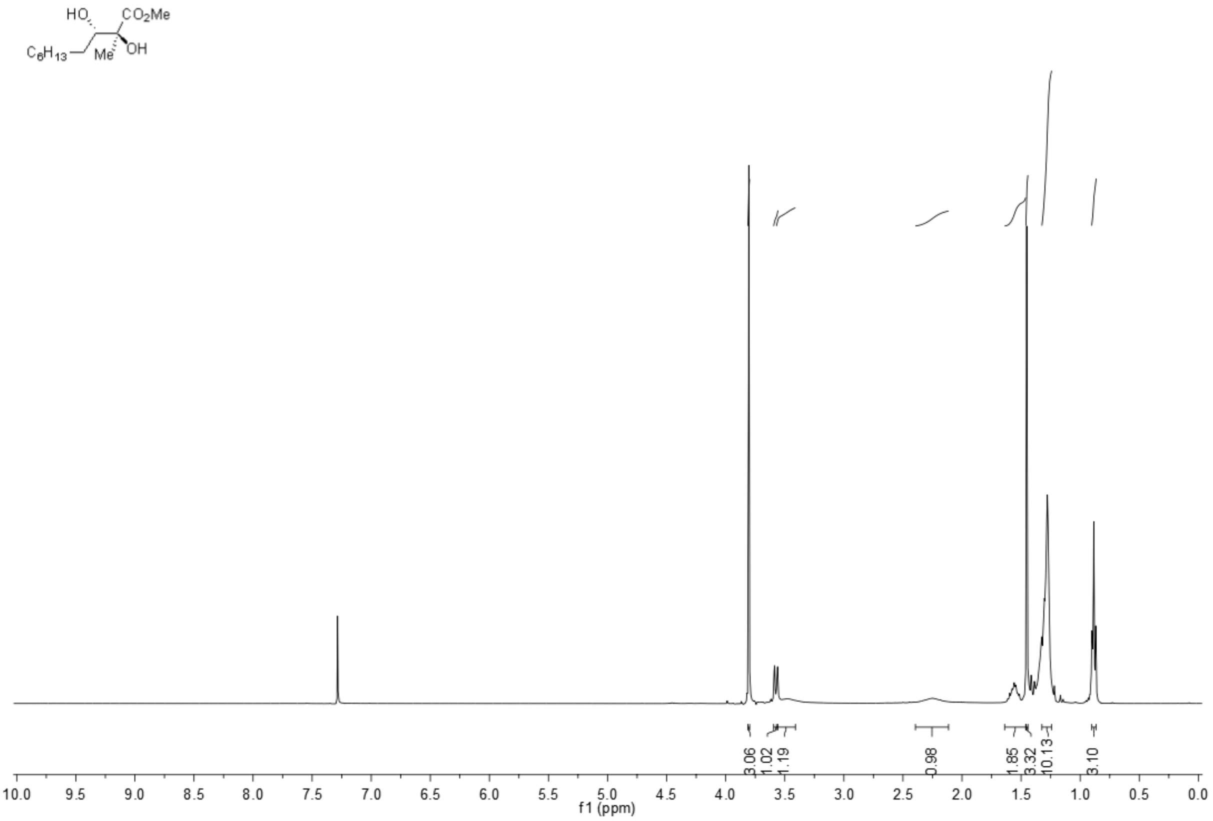

^{13}C-NMR (101 MHz, $CDCl_3$)

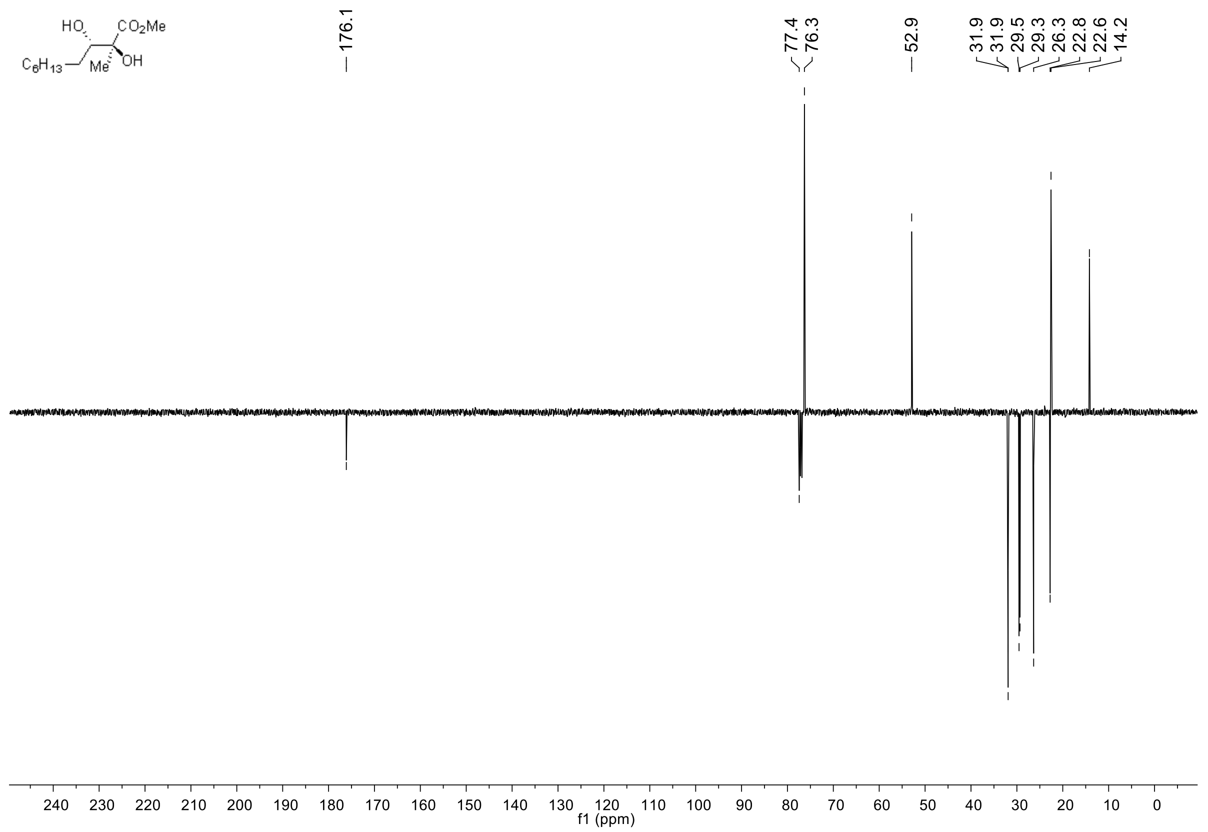

^{1}H-NMR (400 MHz, $CDCl_3$)

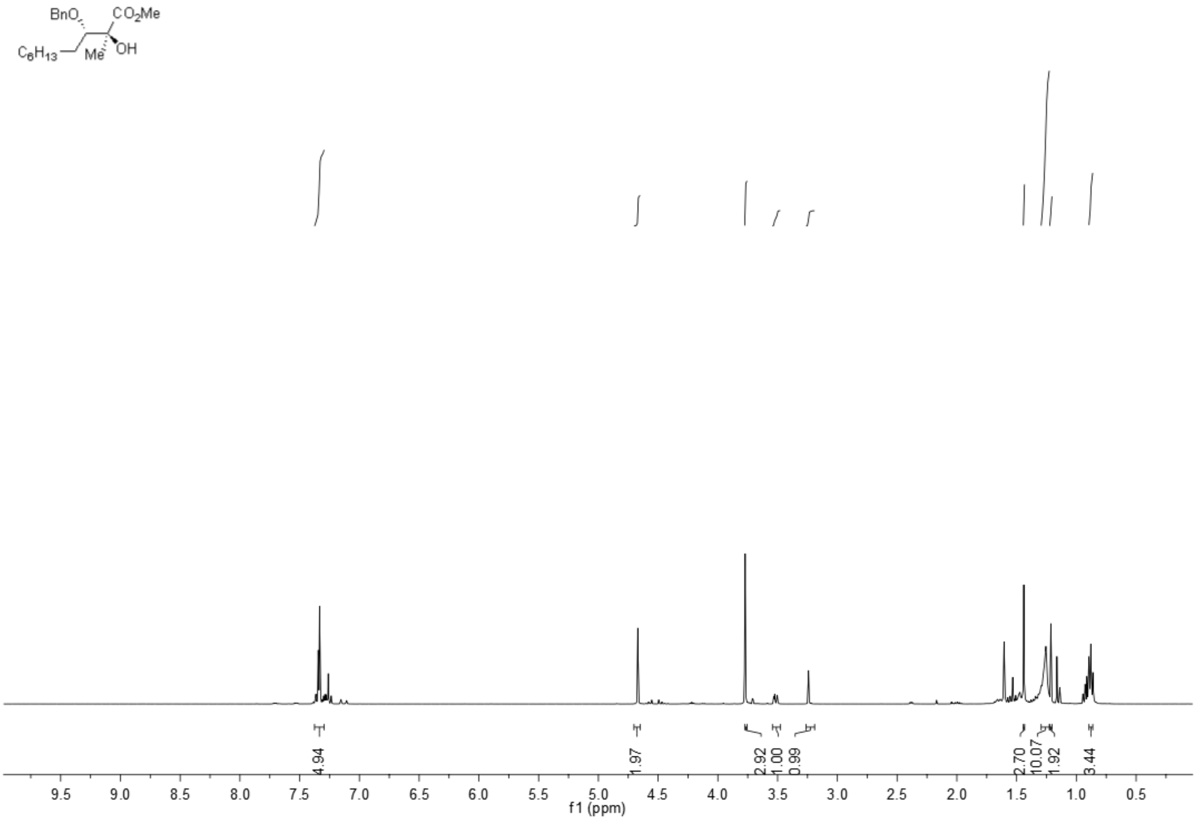

^{13}C-NMR (101 MHz, $CDCl_3$)

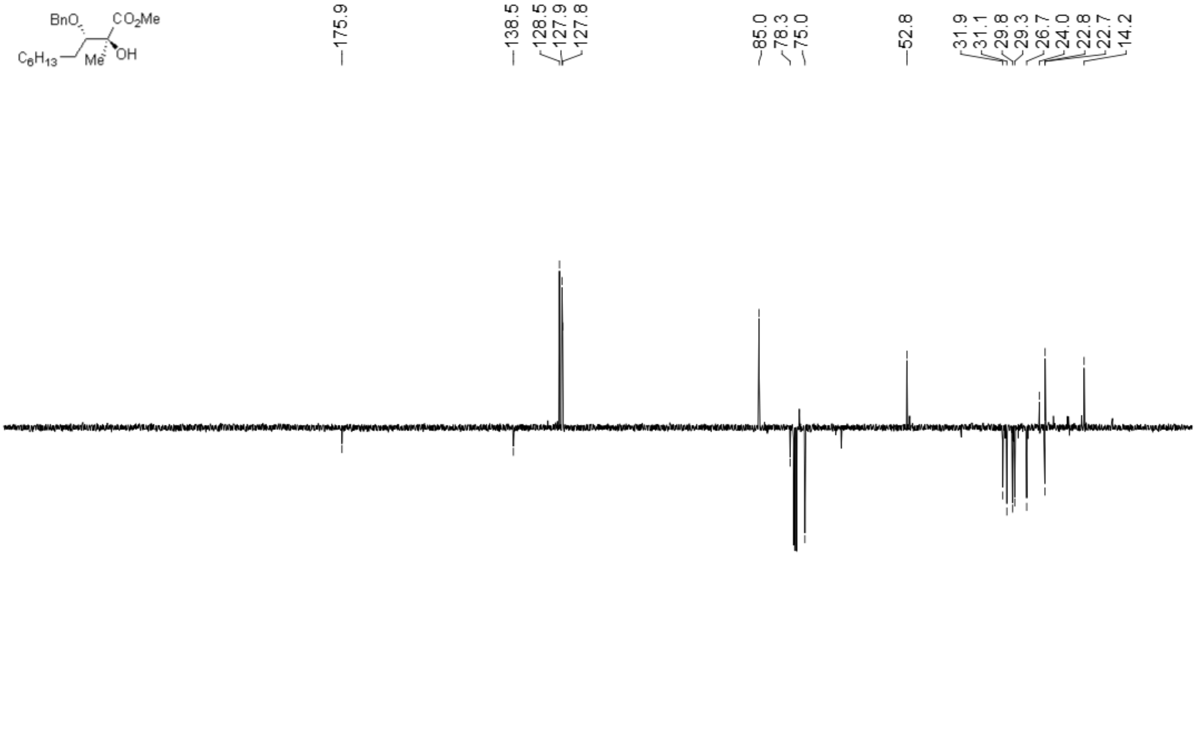

^{1}H-NMR (400 MHz, $CDCl_3$)

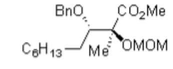

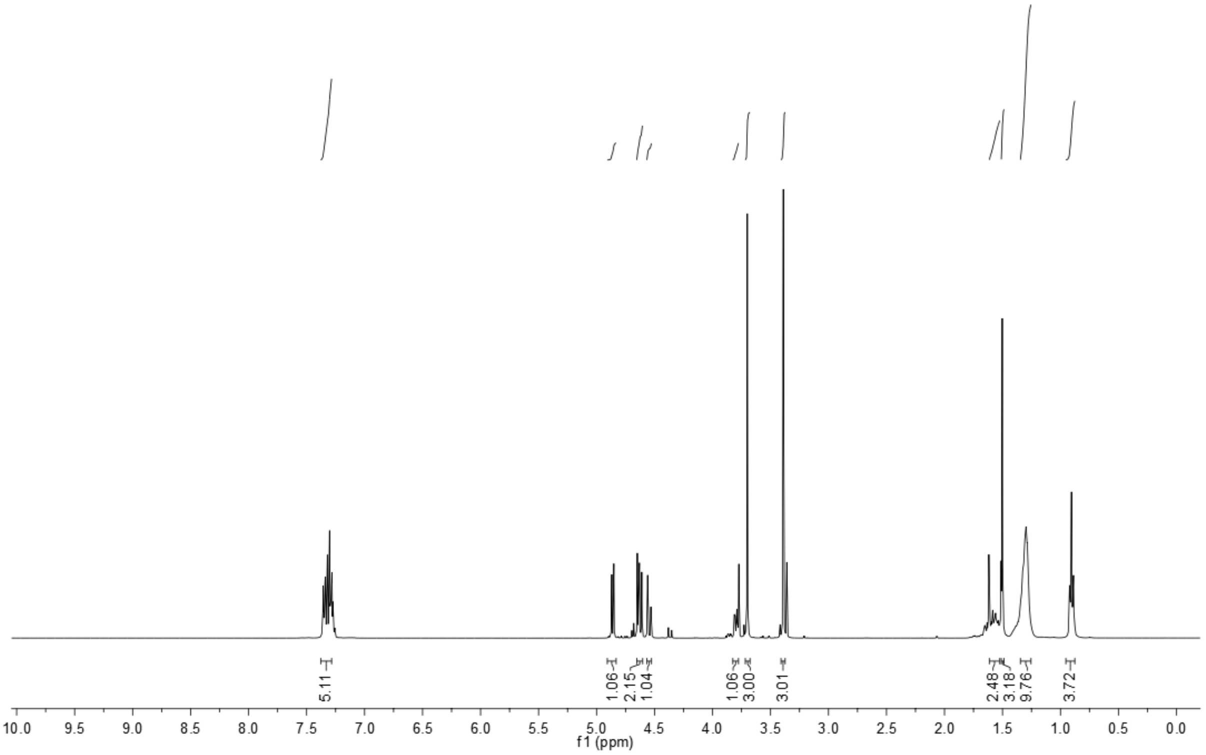

^{13}C-NMR (101 MHz, $CDCl_3$)

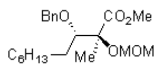

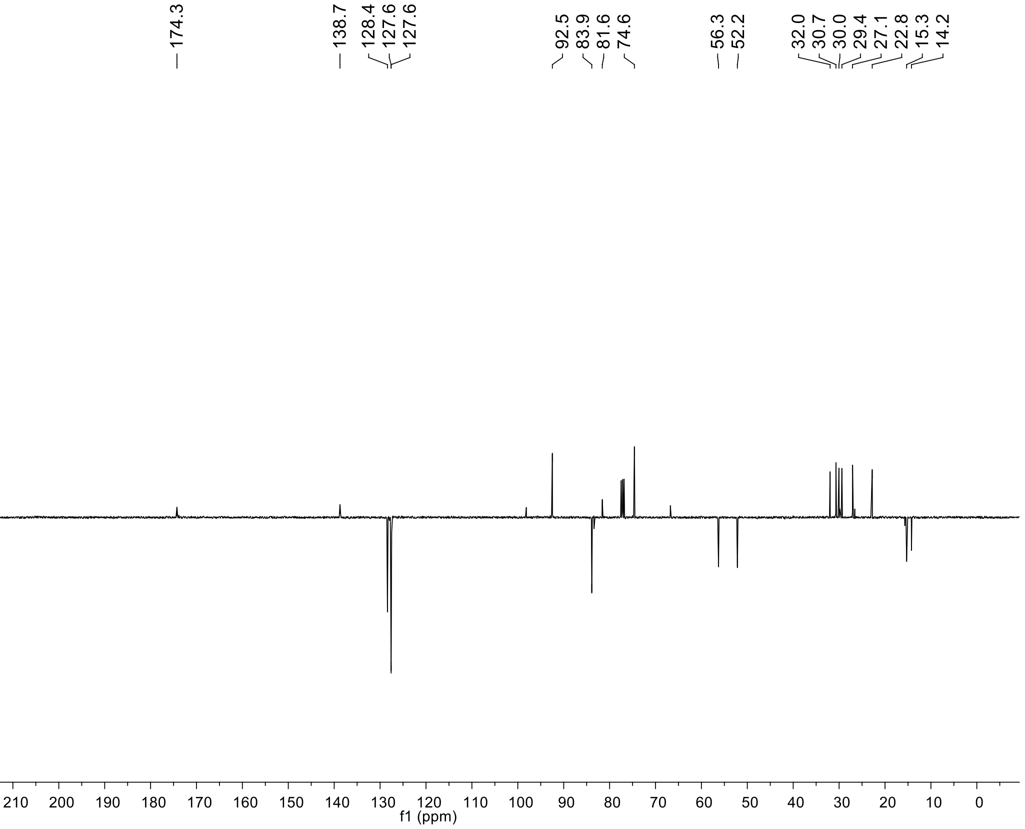

^{1}H-NMR (400 MHz, $CDCl_3$)

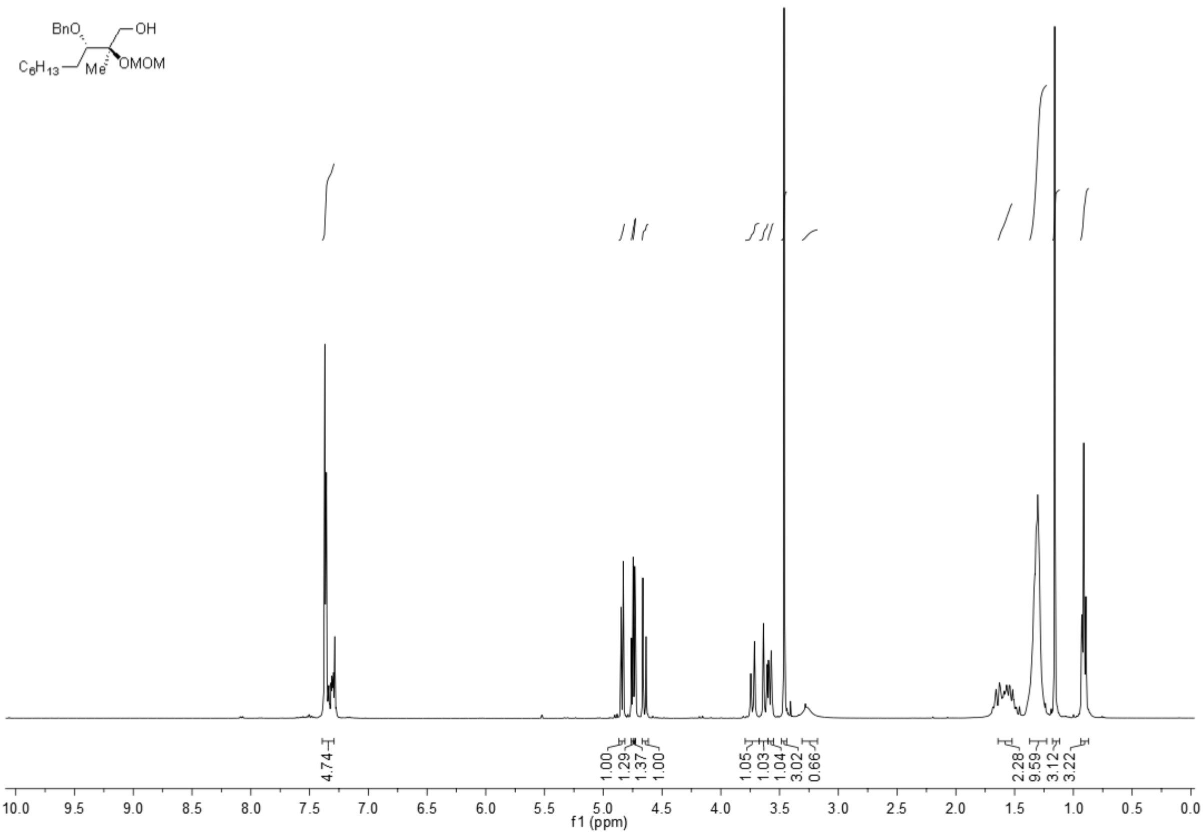

^{13}C-NMR (101 MHz, $CDCl_3$)

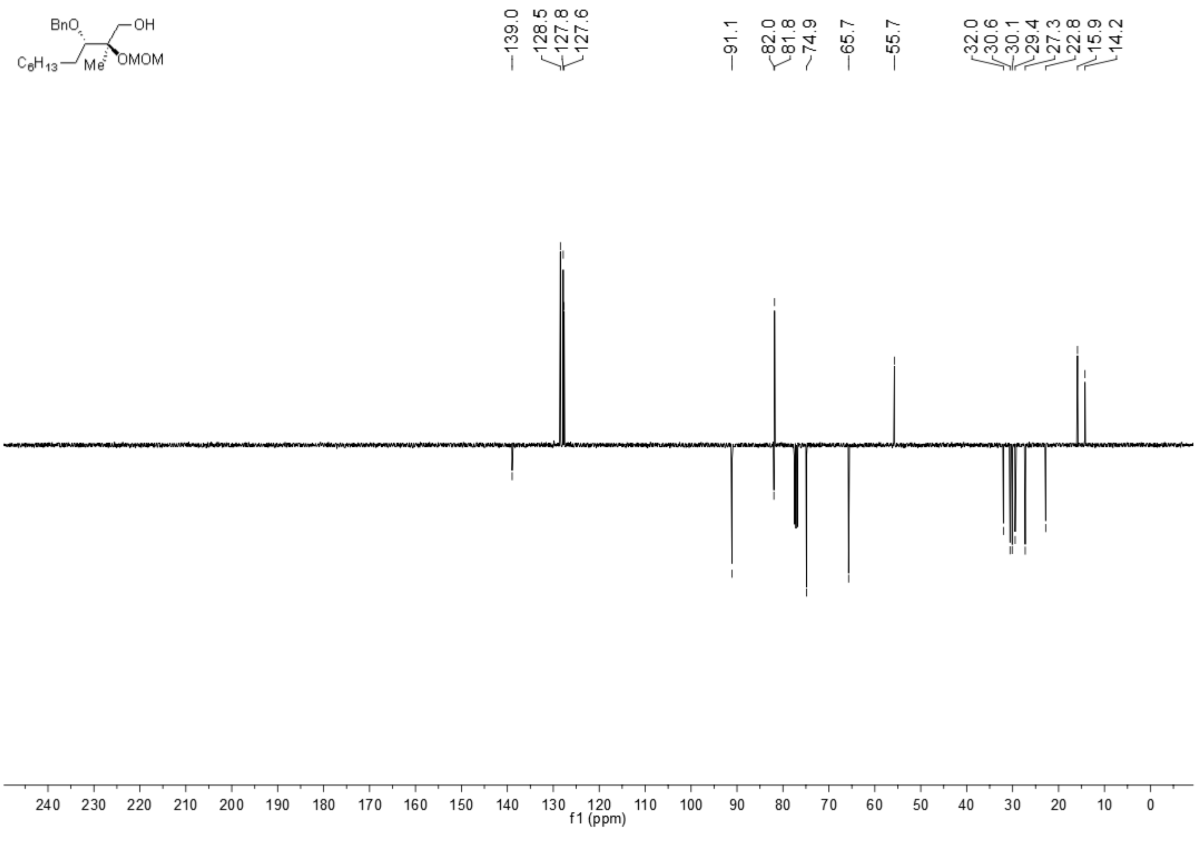

^{1}H-NMR (400 MHz, $CDCl_3$)

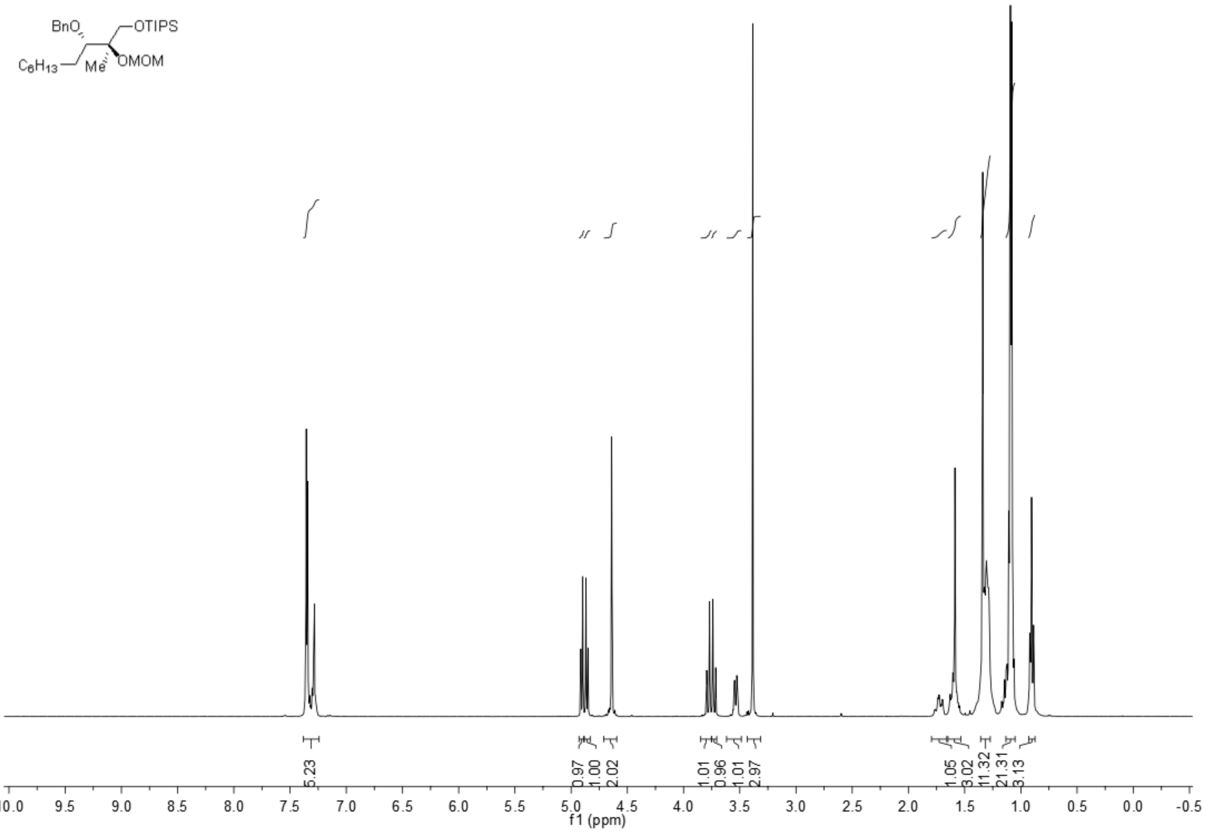

^{13}C-NMR (101 MHz, $CDCl_3$)

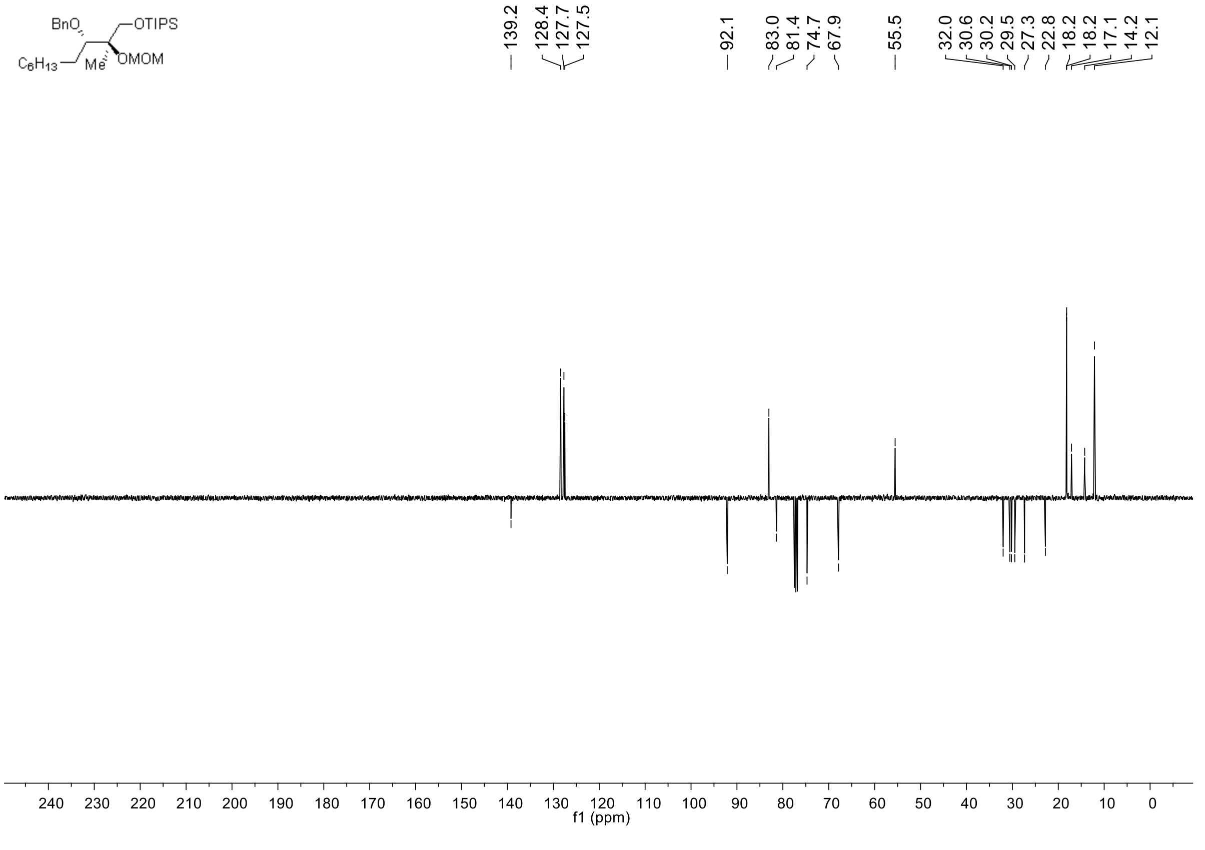

^{1}H-NMR (400 MHz, $CDCl_3$)

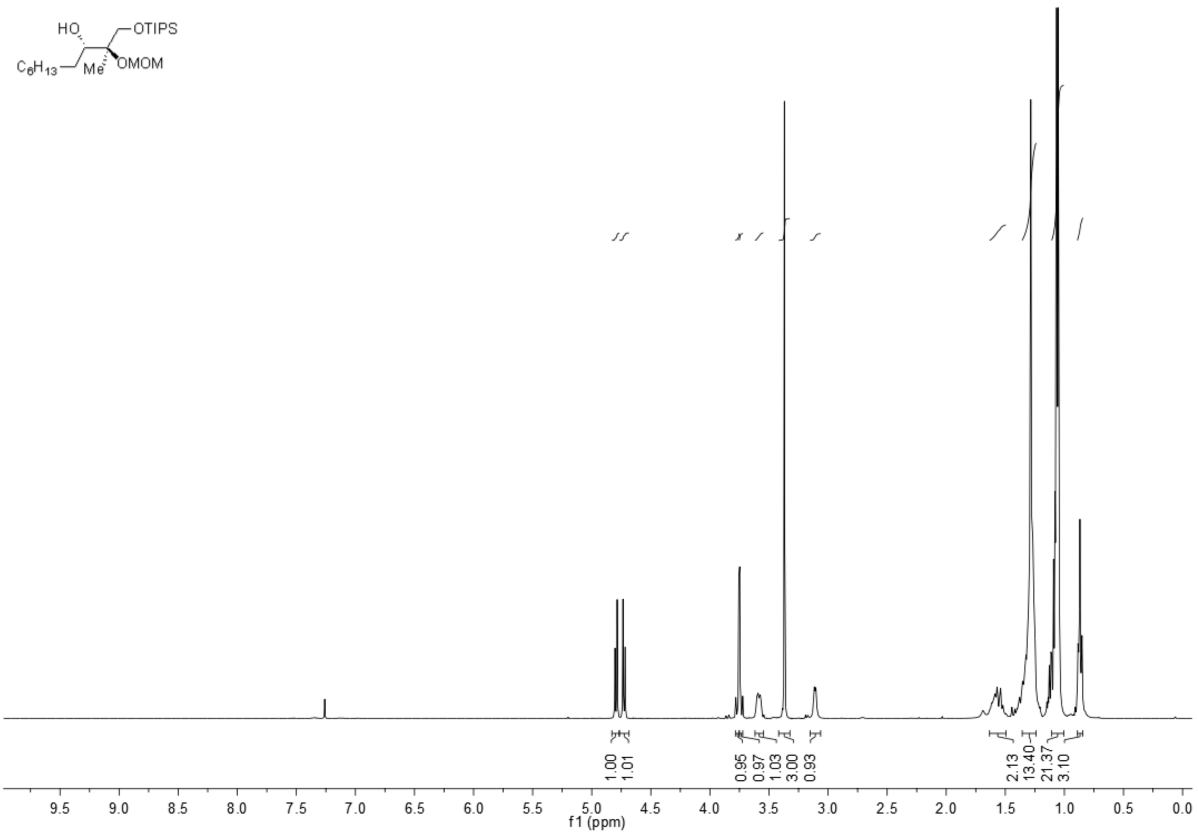

^{13}C-NMR (101 MHz, $CDCl_3$)

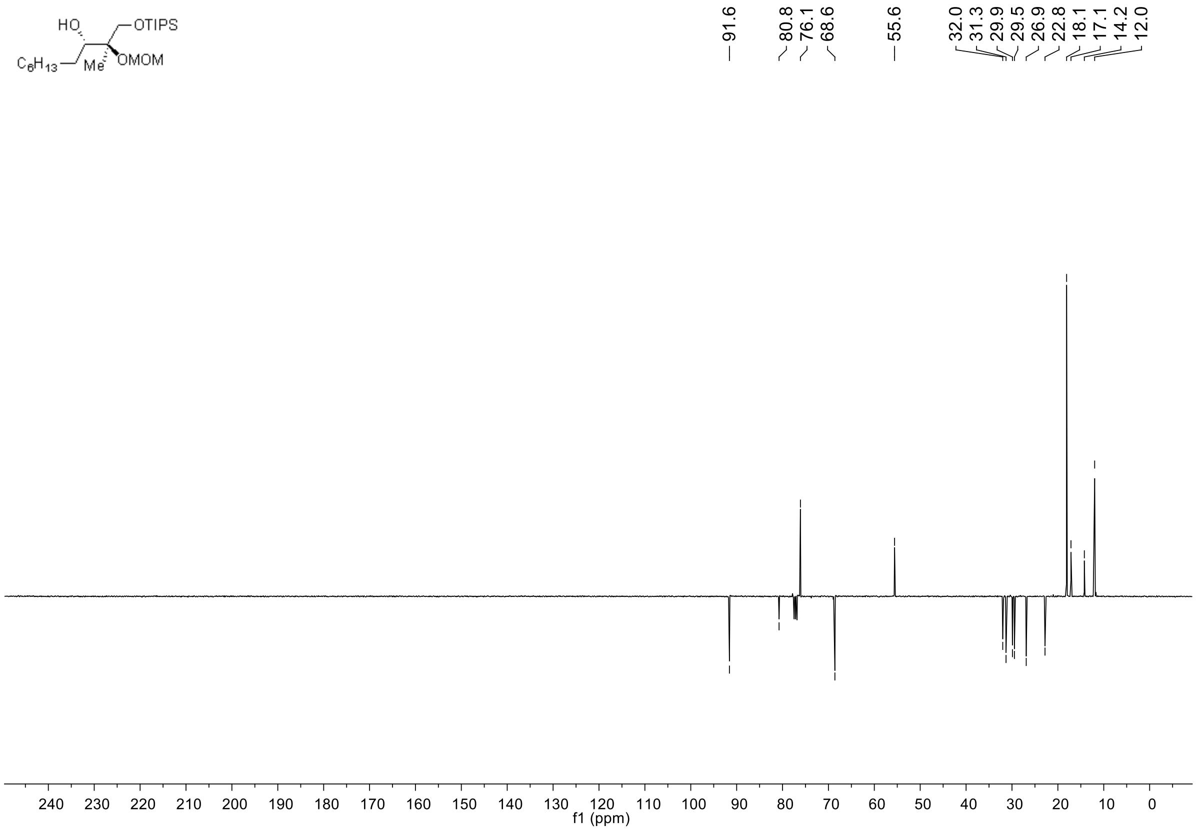

^{1}H-NMR (400 MHz, $CDCl_3$)

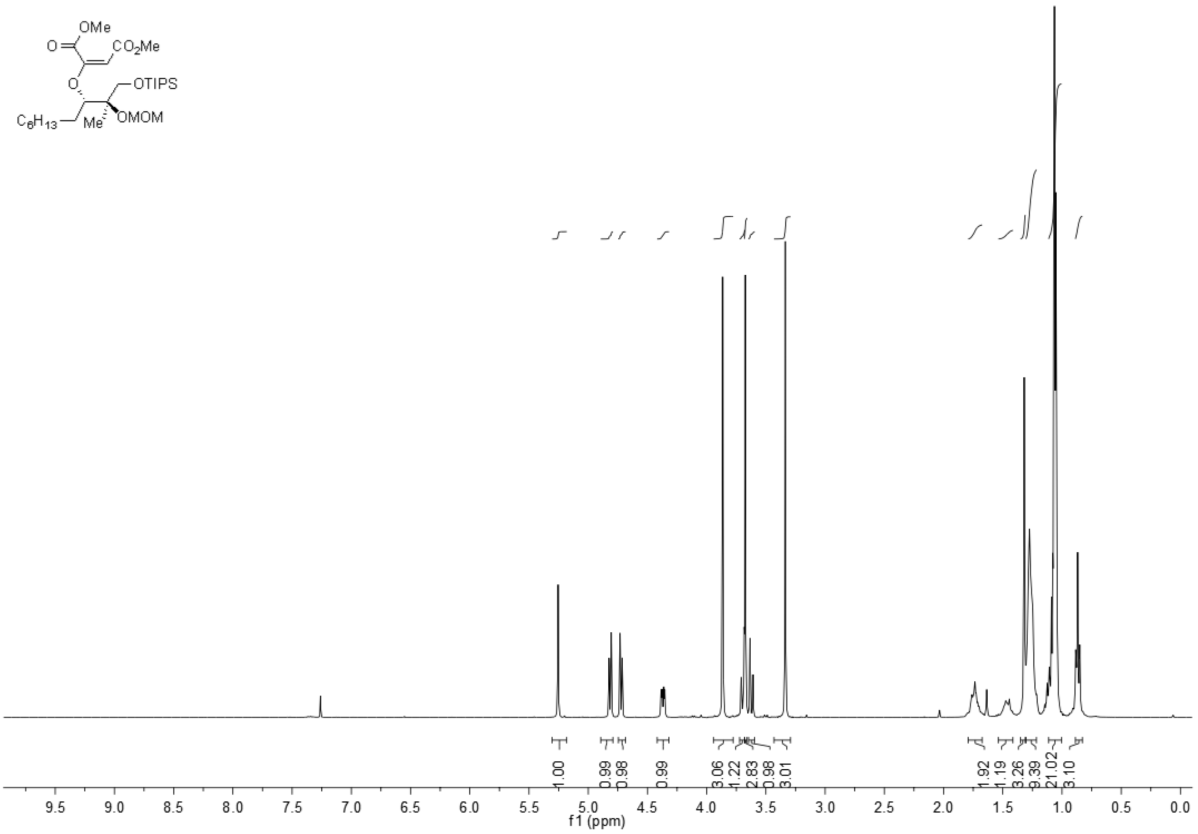

^{13}C-NMR (101 MHz, $CDCl_3$)

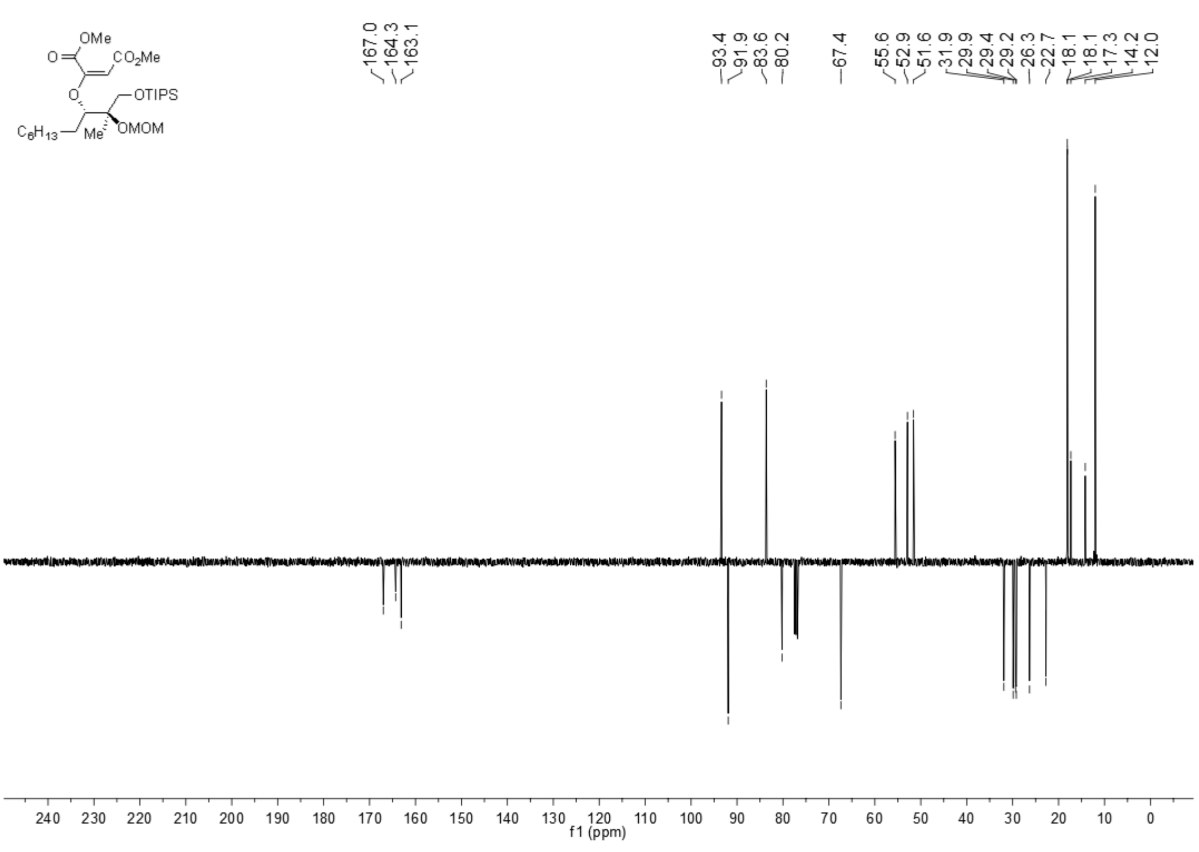

^{1}H-NMR (400 MHz, $CDCl_3$)

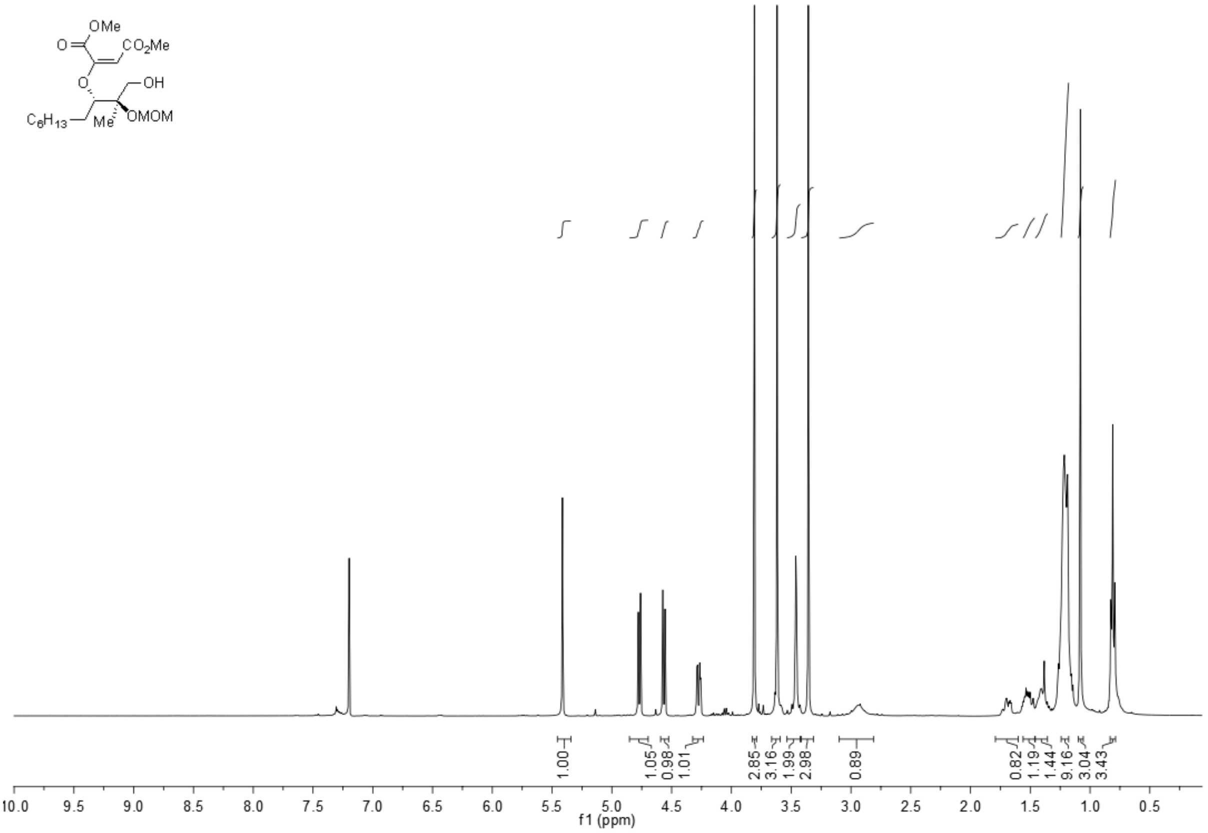

^{13}C-NMR (101 MHz, $CDCl_3$)

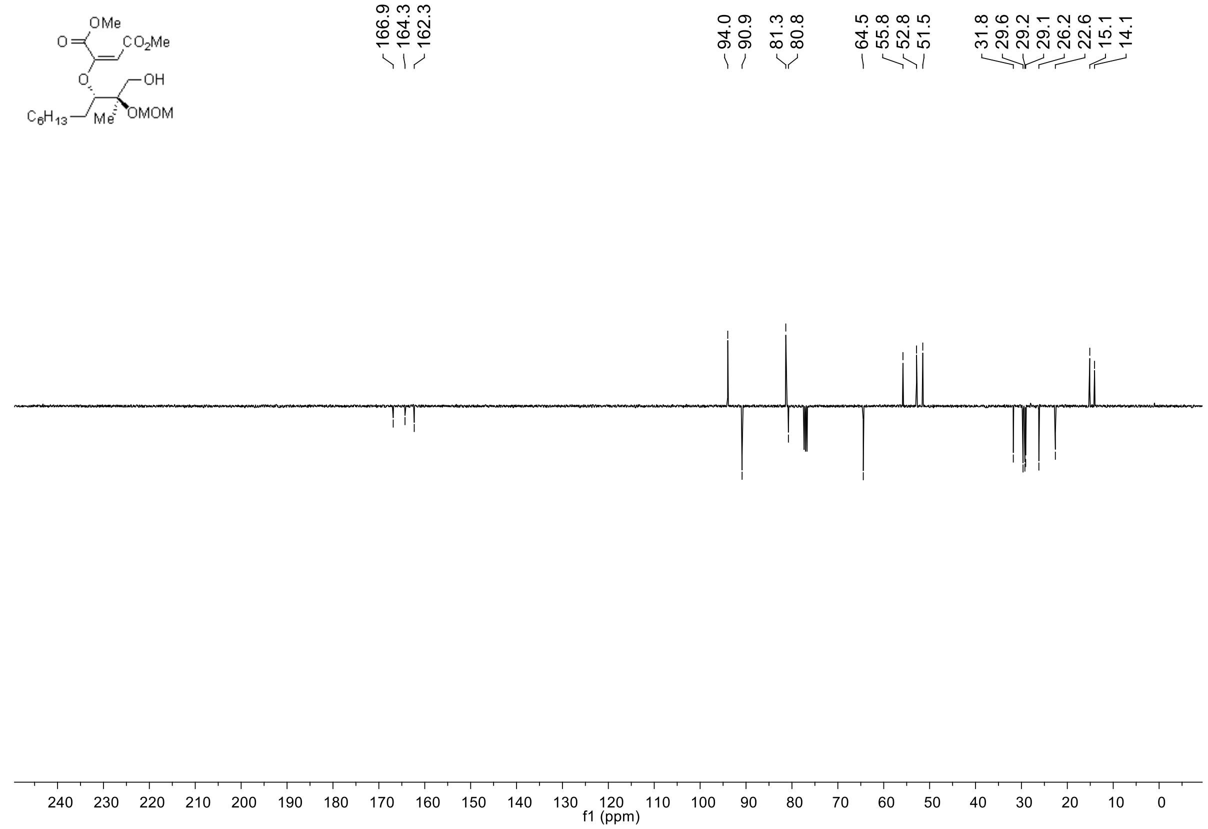

^{1}H-NMR (400 MHz, $CDCl_3$)

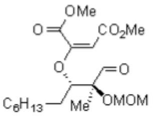

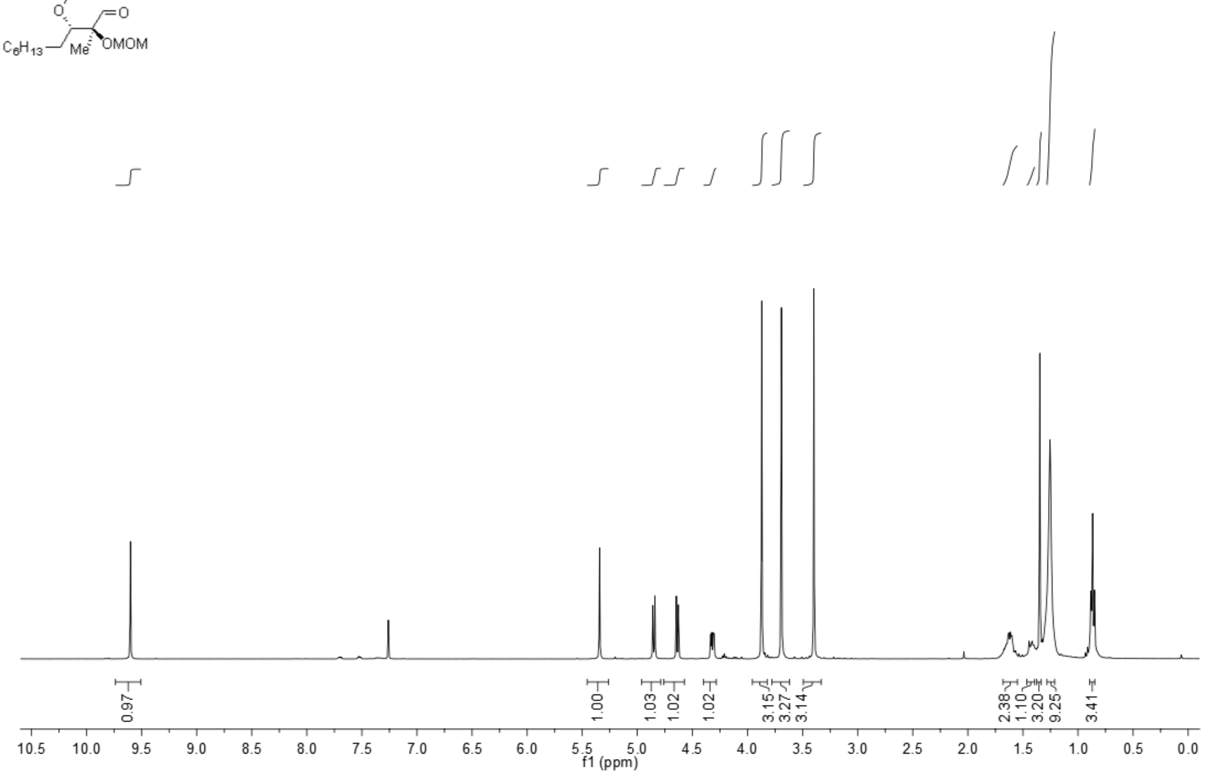

^{13}C-NMR (101 MHz, $CDCl_3$)

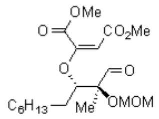

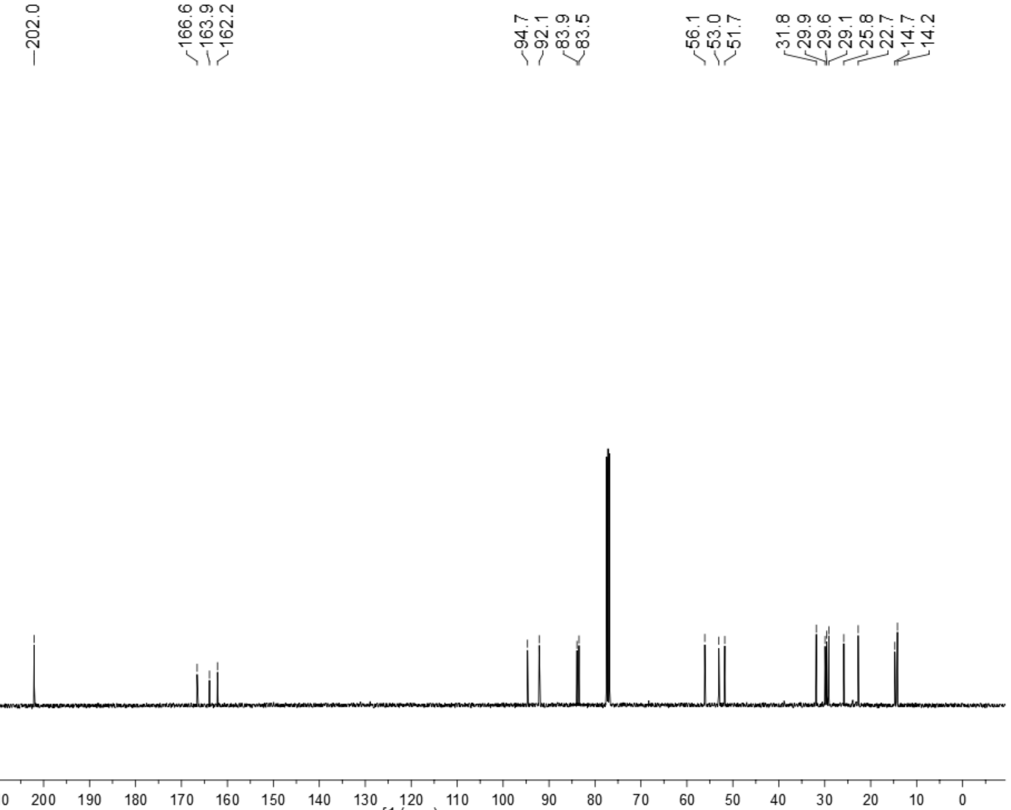

^{1}H-NMR (400 MHz, $CDCl_3$)

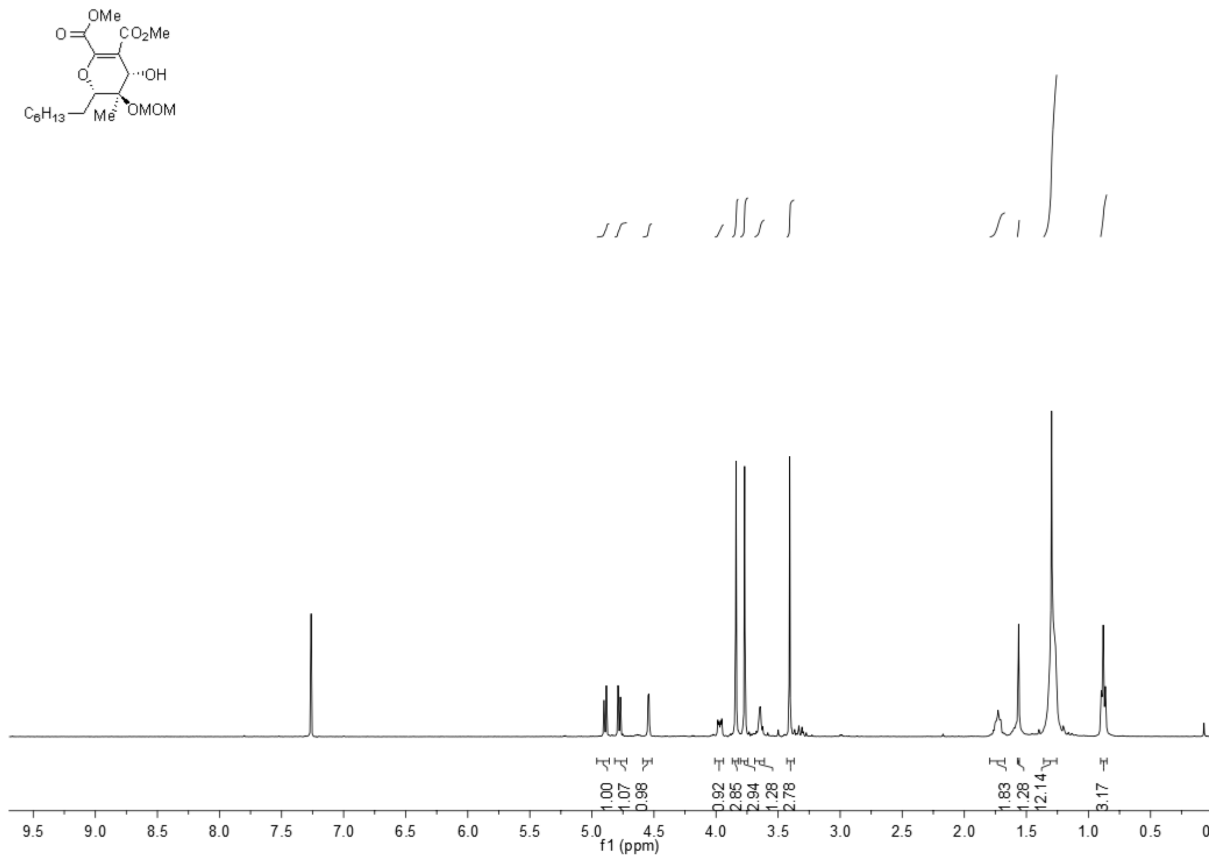

^{13}C-NMR (101 MHz, $CDCl_3$)

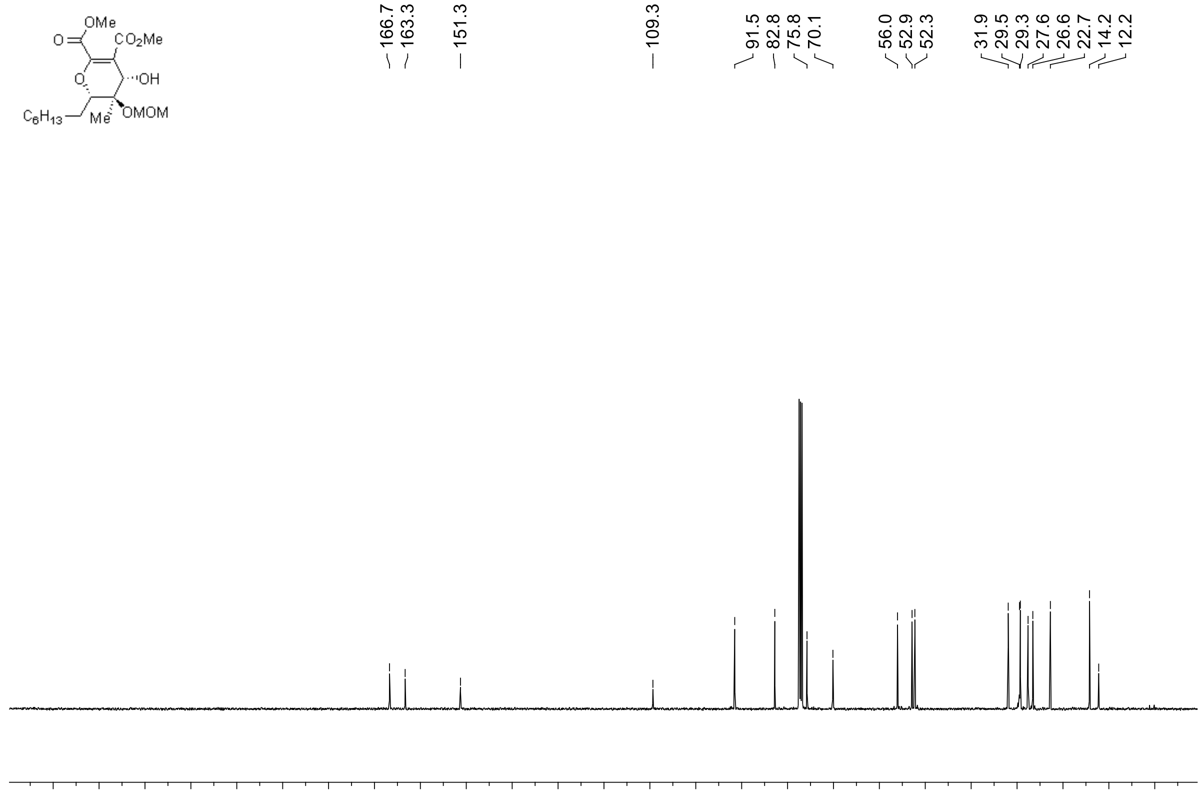

^{1}H-NMR (400 MHz, $CDCl_3$)

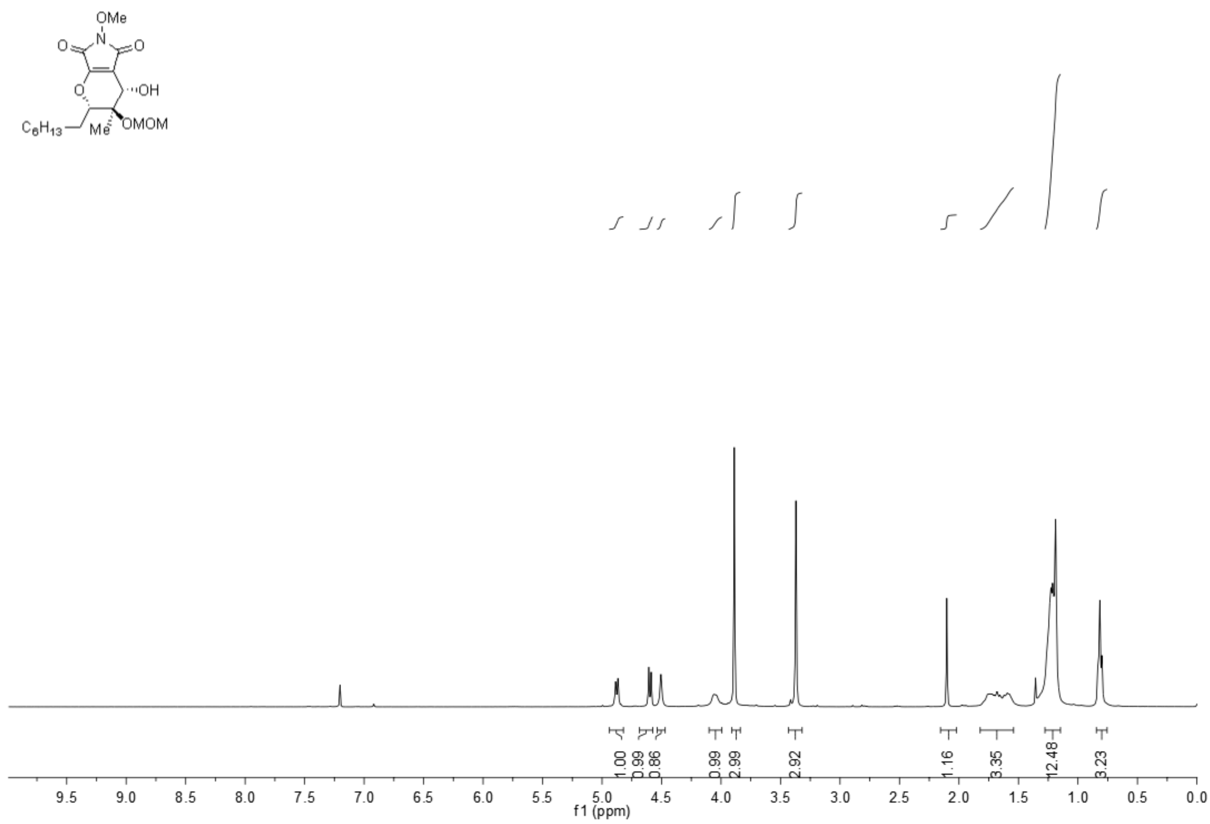

^{13}C-NMR (101 MHz, $CDCl_3$)

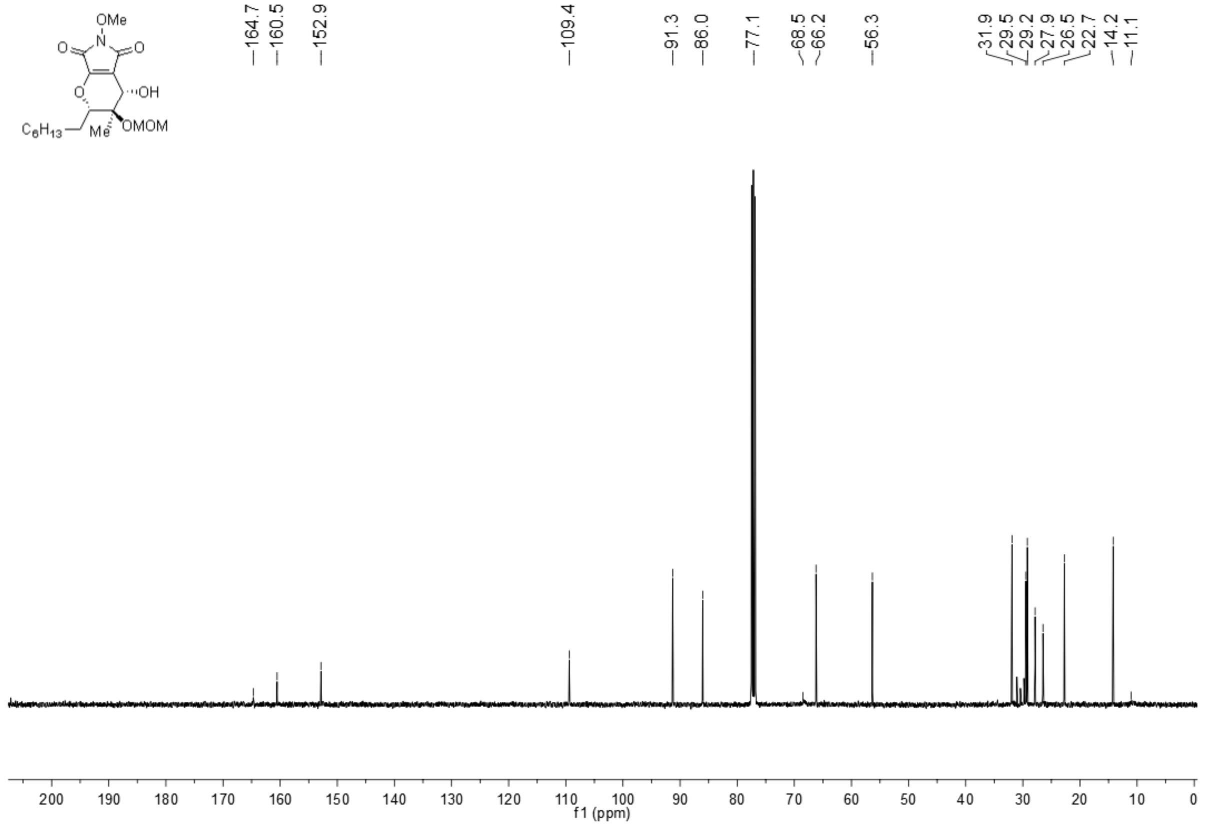

^{1}H-NMR (500 MHz, DMSO-*d6*)

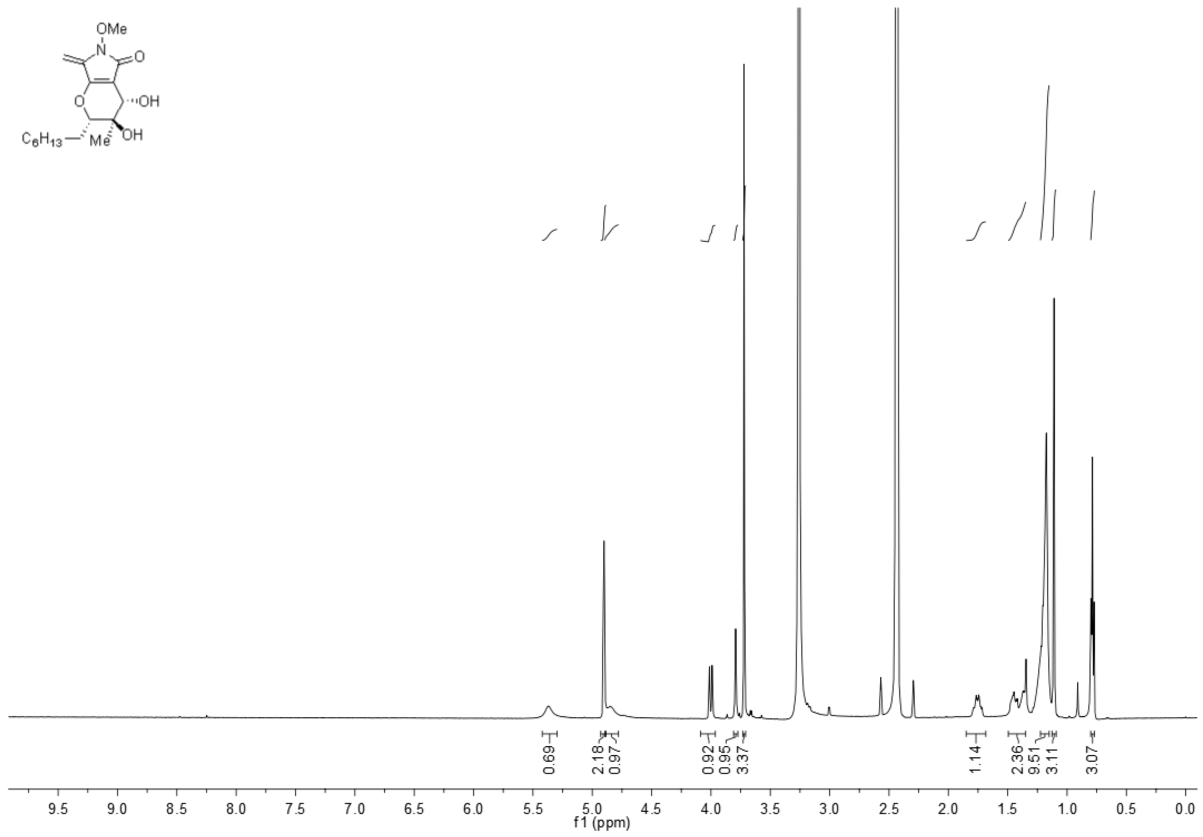

^{13}C-NMR (125 MHz, DMSO-*d6*)

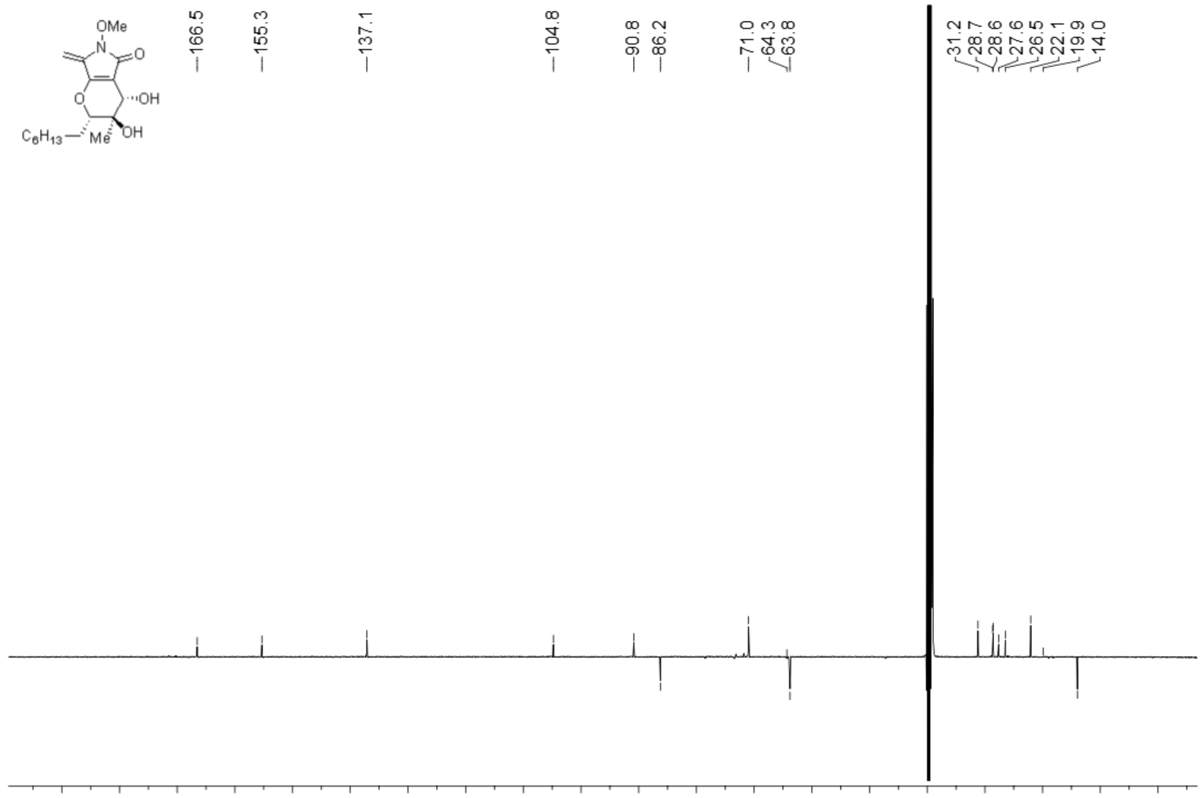

HMBC

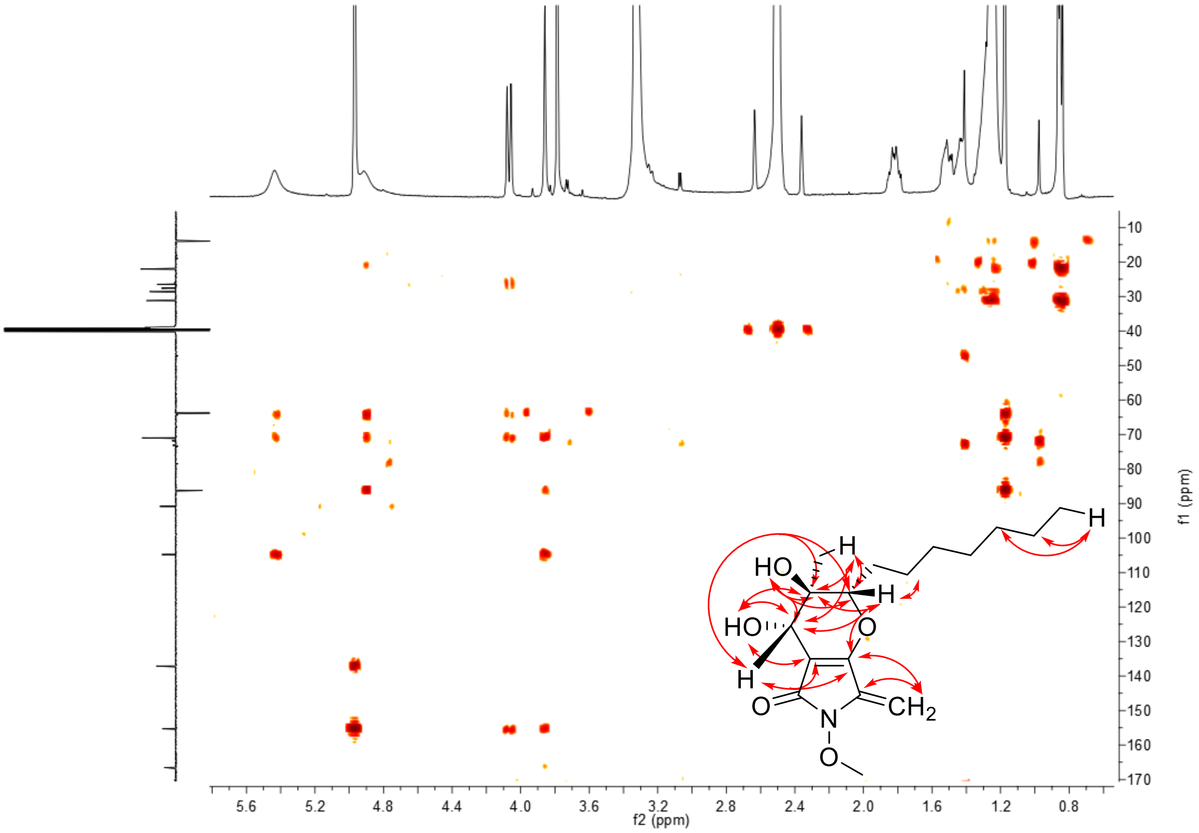

HSQC

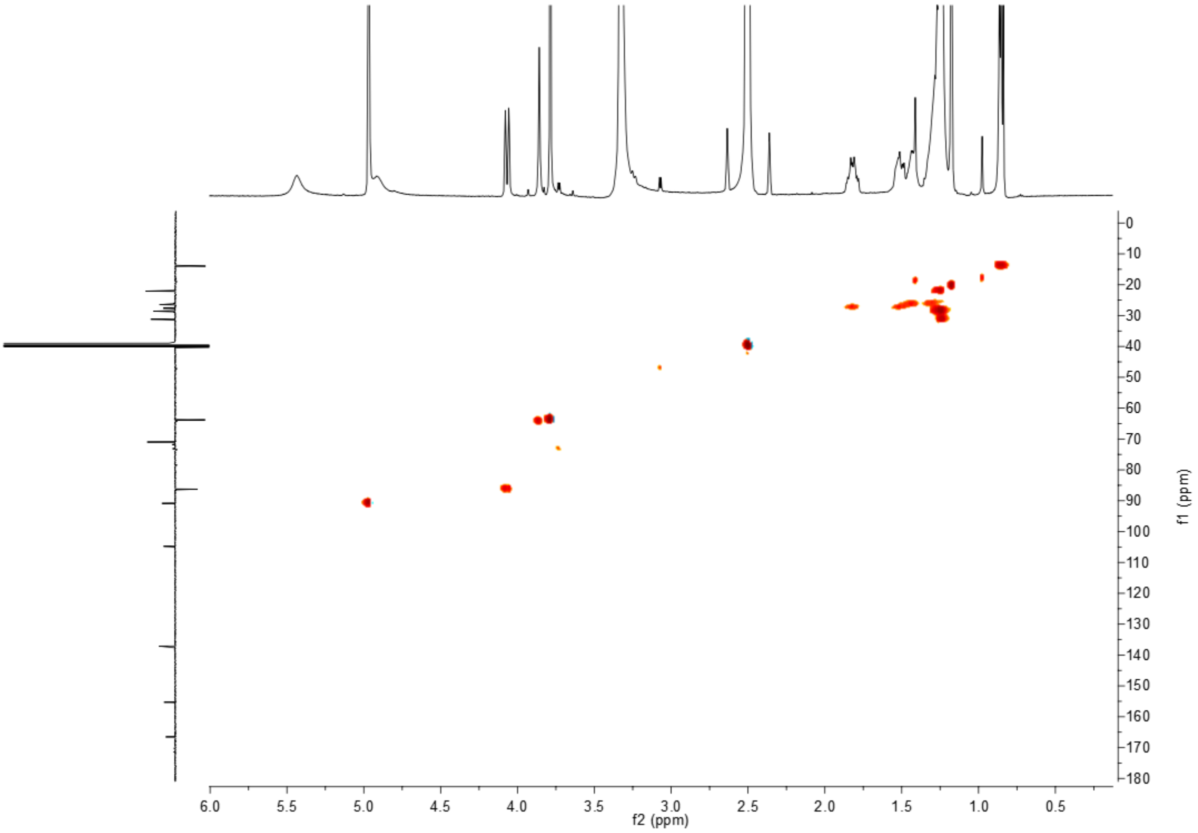

COSY

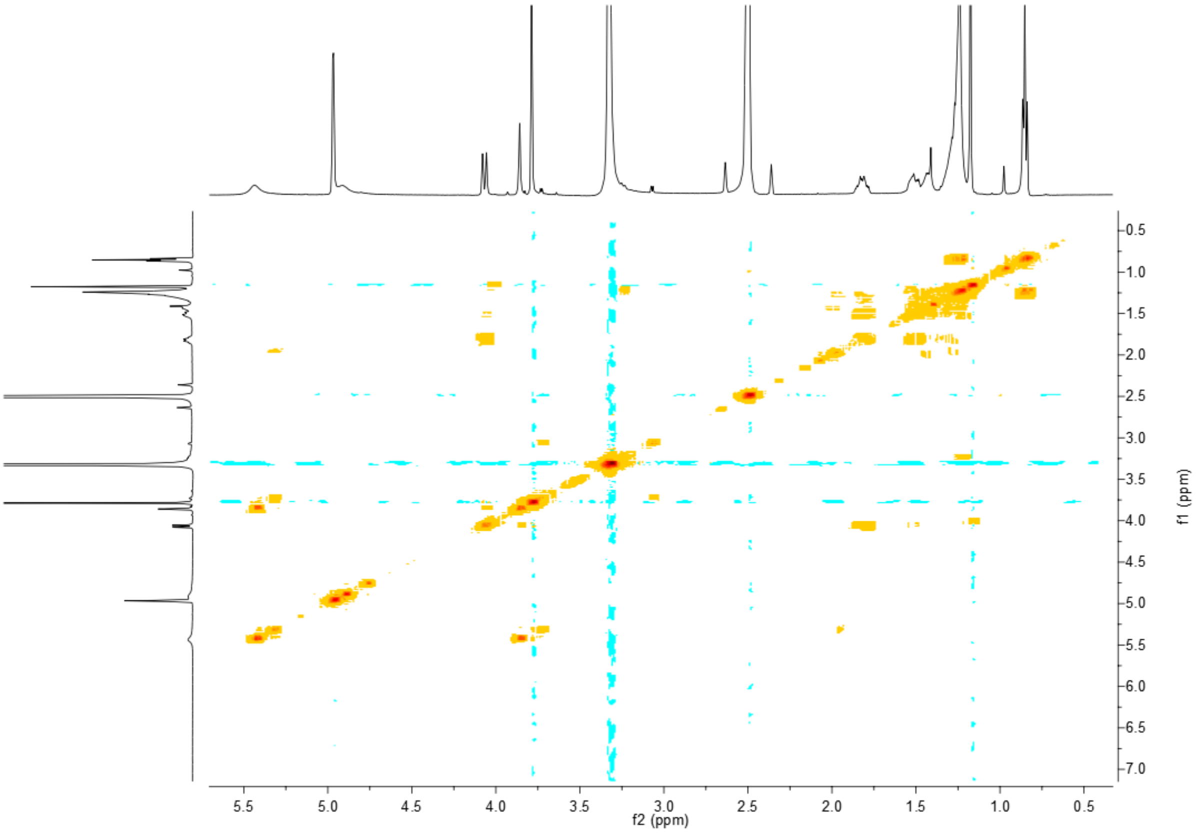
5.5
5.0
4.5
4.0
3.5
3.0
2.5
2.0
1.5
1.0
0.5
f2 (ppm)
0.5
1.0
1.5
2.0
2.5
3.0
3.5
4.0
4.5
5.0
5.5
6.0
6.5
7.0
f1 (ppm)

4. X-ray data for 5

Crystal Data for **5**: $C_7H_{11}NO_4$ (M =173.17 g/mol): monoclinic, space group P2$_1$/n (no. 14), a = 9.7222(3) Å, b = 7.91107(17) Å, c = 11.6743(3) Å, β = 102.086(3)°, V = 878.00(4) Å^3, Z = 4, T = 150(2) K, μ(CuKα) = 0.923 mm^{-1}, $Dcalc$ = 1.310 g/cm^3, 6016 reflections measured (13.31° ≤ 2Θ ≤ 156.668°), 1844 unique (R_{int} = 0.0245, R_{sigma} = 0.0198) which were used in all calculations. The final R_1 was 0.0382 (I > 2σ(I)) and wR_2 was 0.1115 (all data). Data deposited at Cambridge Crystallographic Data Centre: CCDC 1838655. Solid state structure of **5** with thermal ellipsoids drawn at 50% probability level:

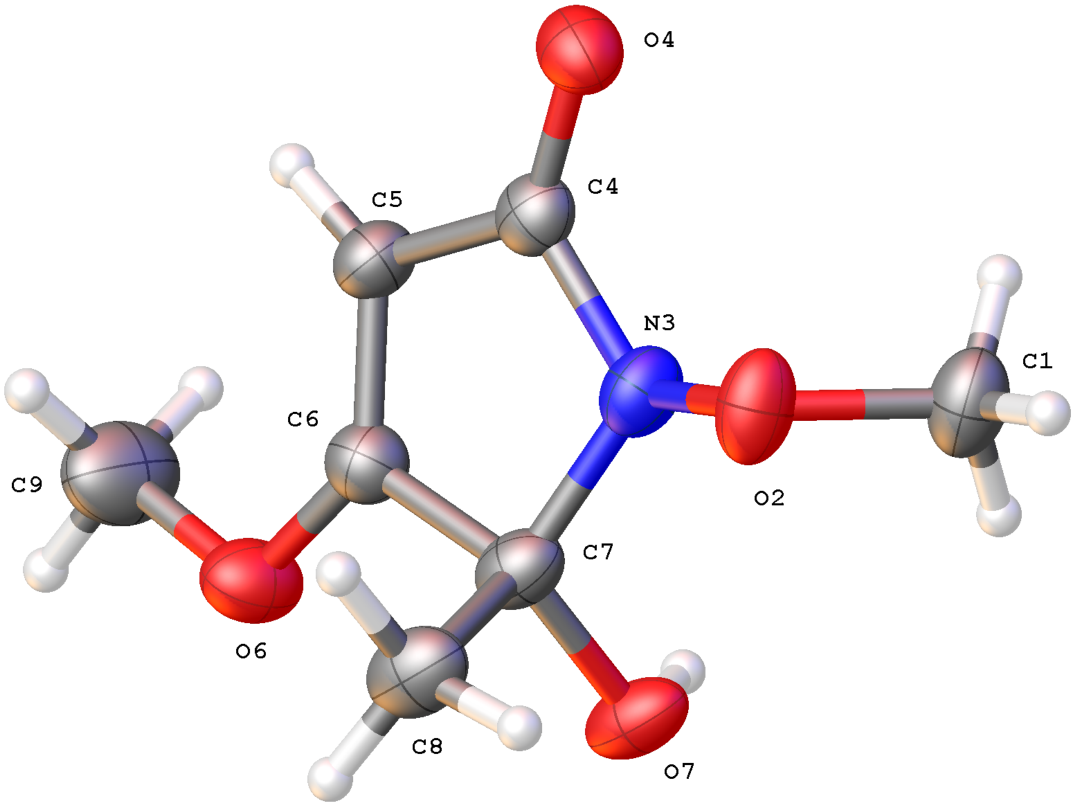

5. Plant growth assays for 1, 4 and synthetic phyllostictine A

The root growth assays were performed with Arabidopsis Col-0 seedlings, ecotype background. After surface sterilization seeds were sown onto square petri dishes containing 30 mL of half-strength Murashige and Skoog medium in 1.5% agar and with 0.5% sucrose. The seeds were stratified in the dark at 4°C for 48 hours before germination for 6 days under lights at 22°C for day and 18°C for night conditions, in 12-hour cycles. Seedlings were then transferred to fresh plates with test compounds incorporated into the agar. The position of the primary root tip was marked and the seedlings were grown for another 6 days, after which the plates were scanned and the elongation of the primary root during treatment was measured using ImageJ 1.51w.

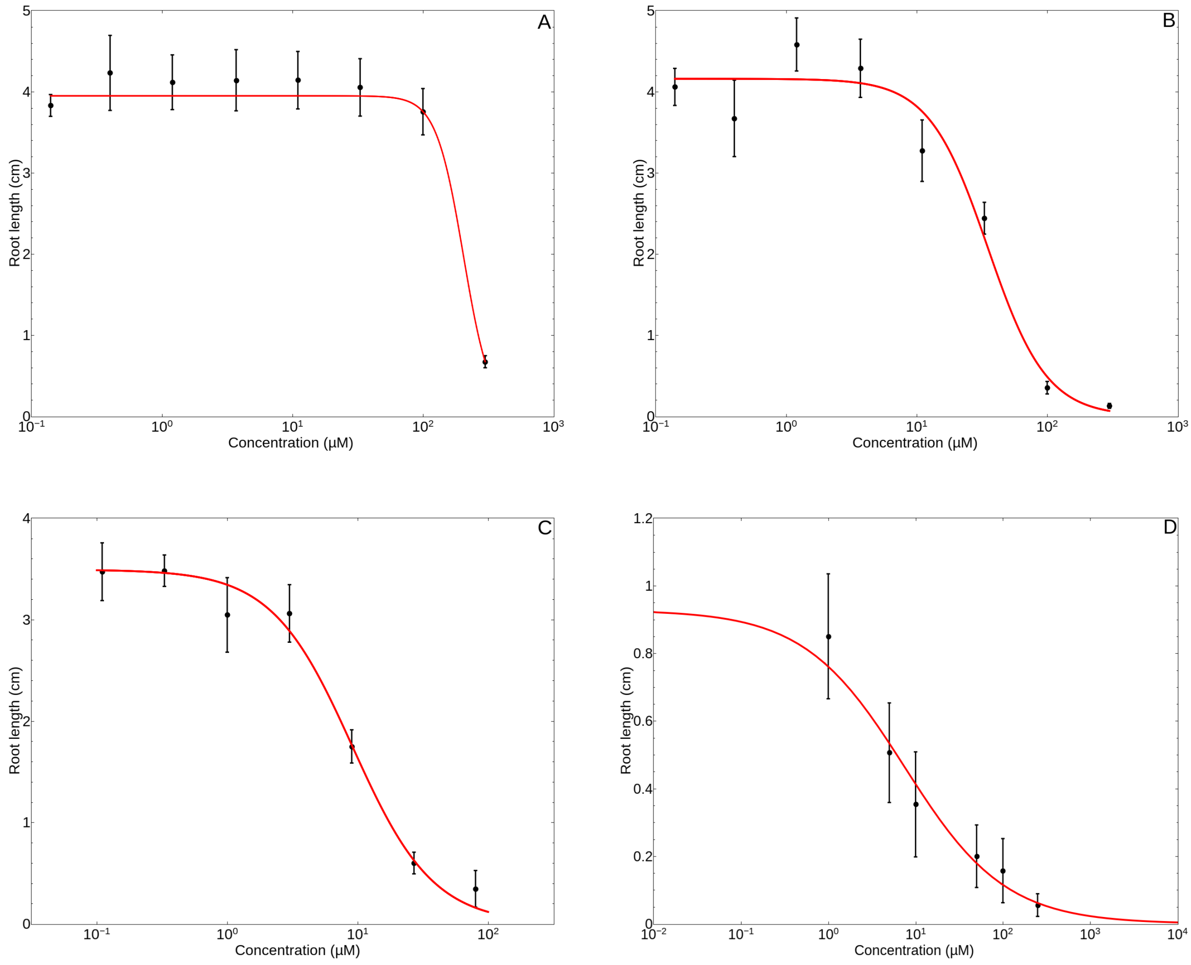

Dose-response curves for root growth inhibition using Arabidopsis seedlings. A: Compound **4** (IC_{50} = 205 ±19 µM). B: Compound 1 (IC_{50} = 35 ±6 µM). C: Phyllostictine A (IC_{50} = 9 ±1 µM). D: Glyphosate (IC_{50} = 7 ±2 µM). In D, a series of control plates were read to establish growth in untreated seedlings and anchor the fit and in this experiment growth was recorded after 4 days. In all the other root growth assays growth was recorded after 6 days. Error bars represent standard deviations of the means and the curves are fitted utilizing the formula y = M/(1+exp(-k*(x-x0))) where y is the root length, x is the logarithm base 10 of the concentration, and the parameters to fit are M, k and x0. M is the maximum root length, k is a parameter inversely related to the width of the transition region, and x0 is the logarithm base 10 of the IC_{50}.

6. References

1. W. J. Croxall and H. J. Schneider, *J. Am. Chem. Soc.*, 1949, **71**, 1257.
2. M.-J. Fan, G.-Q. Li and Y.-M. Liang, *Tetrahedron*, 2006, **62**, 6782.
3. S. Balieu, G. E. Hallett, M. Burns, T. Bootwicha, J. Studley and V. K. Aggarwal, *J. Am. Chem. Soc.*, 2015, **137**, 4398.
4. S. Sano, T. Takehisa, S. Ogawa, K. Yokoyama and Y. Nagao, *Chem. Pharm. Bull.*, 2002, **50**, 1300.
5. T. Nawrath, K. Gerth, R. Mueller and S. Schulz, *Chem. Biodivers.*, 2010, **7**, 2228.
6. V. V. Abzianidze, E. V. Poluektova, K. P. Bolshakova, T. L. Panikorovskii, A. S. Bogachenkov and A. Berestetskiy, *Acta Cryst E.,* 2015, **71**, 625.
